# The SLIM1 transcription factor affects sugar signaling during sulfur deficiency in Arabidopsis

**DOI:** 10.1093/jxb/erac371

**Published:** 2022-09-13

**Authors:** Anna Wawrzyńska, Justyna Piotrowska, Anastasia Apodiakou, Franziska Brückner, Rainer Hoefgen, Agnieszka Sirko

**Affiliations:** Laboratory of Plant Protein Homeostasis, Institute of Biochemistry and Biophysics Polish Academy of Sciences, Warsaw, Poland; Laboratory of Plant Protein Homeostasis, Institute of Biochemistry and Biophysics Polish Academy of Sciences, Warsaw, Poland; Max Planck Institute of Molecular Plant Physiology, Am Mühlenberg 1, 14476, Potsdam-Golm, Germany; Max Planck Institute of Molecular Plant Physiology, Am Mühlenberg 1, 14476, Potsdam-Golm, Germany; Max Planck Institute of Molecular Plant Physiology, Am Mühlenberg 1, 14476, Potsdam-Golm, Germany; Laboratory of Plant Protein Homeostasis, Institute of Biochemistry and Biophysics Polish Academy of Sciences, Warsaw, Poland; Michigan State University, USA

**Keywords:** Anthocyanin, glucose signaling, PAP1/MYB75, SLIM1, sucrose signaling, sulfur deficiency

## Abstract

The homeostasis of major macronutrient metabolism needs to be tightly regulated, especially when the availability of one or more nutrients fluctuates in the environment. Both sulfur metabolism and glucose signaling are important processes throughout plant growth and development, as well as during stress responses. Still, very little is known about how these processes affect each other, although they are positively connected. Here, we showed in Arabidopsis that the crucial transcription factor of sulfur metabolism, SLIM1, is involved in glucose signaling during shortage of sulfur. The germination rate of the *slim1_KO* mutant was severely affected by high glucose and osmotic stress. The expression of SLIM1-dependent genes in sulfur deficiency appeared to be additionally induced by a high concentration of either mannitol or glucose, but also by sucrose, which is not only the source of glucose but another signaling molecule. Additionally, SLIM1 affects *PAP1* expression during sulfur deficiency by directly binding to its promoter. The lack of *PAP1* induction in such conditions leads to much lower anthocyanin production. Taken together, our results indicate that SLIM1 is involved in the glucose response by modulating sulfur metabolism and directly controlling *PAP1* expression in Arabidopsis during sulfur deficiency stress.

## Teaser

During sulfur deficiency, the SLIM1 transcription factor directly and positively regulates *PAP1*/*MYB75*expression to induce sucrose-dependent anthocyanin biosynthesis, and hexokinase1 participates in signaling glucose levels through SLIM1 to transcriptional machinery.

## Introduction

Plants are continuously challenged with environmental variables and therefore need to balance the use of resources between growth processes and adaptation to adverse conditions. One of the most essential compounds in this process is sugar, which has both trophic and signaling activities. Sugar metabolism must be carefully coordinated with environmental cues and different developmental stages (Smeekens *et al.*, 2010; Lastdrager *et al.*, 2014; Sheen, 2014). Most importantly, several sugars are also signaling molecules acting as global regulators, translating nutrient status into transcriptional machinery and thus allowing the plant to coordinate developmental programs with available resources (Eveland and Jackson, 2012). The obvious way to tightly couple metabolism to such regulatory mechanisms is to use metabolic enzymes as active members of transcriptional regulatory complexes. One such example is hexokinase (HXK), which, in addition to glucose phosphorylation in the glycolysis process, acts as a sensor that transduces the sugar availability signal (Harrington and Bush, 2003). Some of the cellular pool of the HXK1 protein is present in the nucleus, where it can be associated with transcriptional complexes (Cho *et al.*, 2006).

Glucose has been reported to be a positive regulator of TARGET OF RAPAMYCIN (TOR) kinase activity (Dobrenel *et al.*, 2013), whose key function is sensing cellular energetic status and nutrient availability. The transcriptional reprogramming of large gene sets involved in central and secondary metabolism, cell cycle, transcription, signaling, and transport is dictated by glucose–TOR signaling to adjust anabolic processes (Xiong *et al.*, 2013). Sulfur is an important nutrient for plants and its status is signaled to the TOR pathway by glucose (Dong *et al.*, 2017). Plants can take up sulfate and assimilate it through a series of energy-dependent reducing steps to produce sulfide, which is subsequently incorporated into cysteine. Cysteine is the source of reduced sulfur for many pathways and its main storage form is the tripeptide glutathione, the main redox regulator of the plant cell (Jobe and Kopriva, 2018). In the *sir1*-*1* mutant with reduced sulfite reductase activity, sulfide availability is diminished, TOR activity is abolished, and glucose content is significantly lower than that in wild-type Arabidopsis (Dong *et al.*, 2017). Interestingly, TOR activity and cell division in the root apical meristem causing the growth arrest phenotype in the *sir1*-*1* mutant could be rescued by an exogenous supply of glucose (Dong *et al.*, 2017). This suggests that TOR signaling is not independently affected by sulfur availability, but rather by glucose energy signaling. Moreover, blocking γ-glutamyl-cysteine ligase activity, catalysing the first step of glutathione synthesis, partially recovers the dwarf phenotype of the *sir1-1* mutant while increasing TOR activity, suggesting that reallocating sulfur flux from glutathione production to protein translation can enhance plant growth via the regulation of TOR (Speiser *et al.*, 2018). Similarly, genome transcriptome profiling revealed that glucose–TOR signaling regulates the transcription of various genes involved in the transport and metabolism of sulfate (Xiong *et al.*, 2013). Interestingly, 3-hydroxypropylglucosinolate, a plant sulfur-containing defense-related secondary metabolite, acts as a TOR inhibitor, blocking glucose–TOR-promoted root meristem activation and root elongation (Malinovsky *et al.*, 2017). Therefore, the direction of sulfur flux and its metabolites appears to play a critical role in balancing plant growth and stress responses by TOR activity control.

There is a positive correlation between sulfate availability and soluble sugar level in Arabidopsis plants. Sulfate deprivation results in diminished glucose content, whereas short-term sulfide fumigation causes fast and significant upregulation of glucose level (Dong *et al.*, 2017). However, the addition of glucose to the growth medium has a positive effect on the expression of sulfur metabolism genes. After just 6 h of incubation of Arabidopsis seedlings in 6% glucose, all genes encoding enzymes of the assimilatory sulfate reduction pathway show an over 3-fold increase in transcript level (Miao *et al.*, 2016; Nguyen *et al.*, 2019). Adenosine 5ʹ-phosphosulfate reductase (APR), a key enzyme of sulfate reduction in plants, is subject to coordinated metabolic control by carbon metabolism. The addition of 0.5% (w/v) glucose to the medium of Arabidopsis plants kept for 38 h in the dark leads to an increase in APR levels in roots (mRNA, protein, and activity) (Hesse *et al.*, 2003). This result demonstrates that exogenously supplied glucose can replace the function of photoassimilates in the roots, but it also proves that sulfate assimilation efficiency depends on carbon metabolism (Hesse *et al.*, 2003). This phenomenon also translates into sulfur compound levels. Arabidopsis plants accumulate more cysteine and glutathione when they are supplied with glucose both at low (100 μM) and at high (3000 μM) sulfate concentrations (Miao *et al.*, 2016; Nguyen *et al.*, 2019). Glucose also promotes the synthesis of secondary sulfur-containing metabolites in Arabidopsis. Both aliphatic and indolic glucosinolate levels are enhanced without reducing primary sulfur assimilation when plants are cultivated in the presence of glucose (Miao *et al.*, 2013, 2016). The expression of crucial transcription factors regulating the biosynthesis of aliphatic and indolic glucosinolates, namely MYB28 and MYB29, and MYB34 and MYB51, respectively, is enhanced after feeding glucose to the plants. The abolition of that effect in the GLUCOSE-INSENSITIVE2 (*gin2-1*) mutant, which is defective in HXK1 function, together with a substantial reduction of glucosinolate level, indicates an interaction between HXK1 and MYB factors in the regulation of glucosinolate biosynthesis. Glucose-specific induction of glucosinolate accumulation is also evident from the fact that fructose and mannose are ineffective in the stimulation of glucosinolate synthesis (Miao *et al.*, 2013).

The rising awareness of the importance of proper plant sulfur nutrition has resulted in substantial growth in our understanding of sulfur metabolic pathways and their regulatory elements. However, there are many issues to clarify how sulfur metabolism is regulated in response to the changing supply and the internal demand given the plant developmental program and environmental conditions. SULFUR LIMITATION1 (SLIM1), an ethylene insensitive 3-like (EIL) family transcription factor, plays a central role in inducing transcriptional responses whenever more sulfate is needed (Maruyama-Nakashita *et al.*, 2006). EIL family proteins are encoded by six genes in the Arabidopsis genome, with EIN3 being the best-known member (Guo and Ecker, 2004). EIN3, together with its functional homologs EIL1 and EIL2, regulates ethylene-responsive gene expression (Solano *et al.*, 1998). Glucose adversely affects the stability of EIN3 via HXK1, although the molecular link between glucose sensing and EIN3 degradation has not been clarified (Yanagisawa *et al.*, 2003). Very recently, it was demonstrated that EIN3 influences glucose level by contributing to the modulation of cytosolic invertase (CINV) activity (Meng *et al.*, 2020). The major neutral CINV constitutes a key point of control for plant growth. The authors showed that EIN3 binds directly to the promoters of both *PRODUCTION OF ANTHOCYANIN PIGMENT1* (*PAP1*/*MYB75*) and *PHOSPHATIDYLINOSITOL MONOPHOSPHATE 5-KINASE 9* (*PIP5K9*), repressing and enhancing their expression, respectively. In turn, PAP1 binds directly to the promoter of CINV1 and induces its expression, while PIP5K9 interacts with and inhibits CINV1. CINV1 hydrolyses sucrose, releasing the glucose signal, which negatively regulates the stability of EIN3 via HXK1, thereby allowing further accumulation of CINV1. This regulatory mechanism participates in the synchronization of sucrose catabolism with growth and development requirements.

PAP1 is also a well-described positive regulator of anthocyanin biosynthesis in response to sugar and light signaling (Teng *et al.*, 2005). It is well established that sucrose is a strong inducer of anthocyanin in a variety of plant species (Kwon *et al.*, 2011). Sucrose concentrations over a certain threshold act as a signal to the plant that it is actively photosynthesizing. In order to protect from harmful UV irradiation, this leads to the production of protective compounds for photosynthetic pigments, such as anthocyanin. These photoprotective screens also appear in response to different stressors, including nutrient depletion, with the major stimulator of anthocyanin synthesis being PAP1 (Lillo *et al.*, 2008). Though synthetized in the cytosol, anthocyanins do not show their brilliant colors until they are accumulated in the acidic vacuoles (Petrussa *et al.*, 2013). Glutathione directly participates in this process because anthocyanin glutathionylation is crucial for their transport as evidenced by the inability of glutathione *S*-transferase mutants to accumulate anthocyanin in vacuoles (Koes *et al.*, 2005). The ethylene signaling pathway has a negative impact on sucrose-induced anthocyanin accumulation. The *PAP1* transcript level is increased more than 2-fold in a double *ein3eil1* mutant when compared with wild-type seedlings resulting in anthocyanin over-accumulation (Jeong *et al.*, 2010).

To further understand the role of SLIM1 in plant metabolism during sulfur deficiency we created a novel *slim1_KO* mutant using CRISPR/Cas9 technology. In the current study, we investigated the involvement of SLIM1 in plant responses to glucose exposure and its significance in the glucose signaling pathway especially during sulfur deficiency conditions by performing phenotypic, molecular, and genetic studies of the *slim1_KO* mutant, which appeared defective in glucose responses. We also observed the altered response of this mutant to another signaling sugar, sucrose. Furthermore, we investigated a possible molecular target of the cross-talk between sulfur-deficiency and sucrose signaling pathways and demonstrated that SLIM1 targets the *PAP1* promoter to positively regulate its expression. These data support and extend the crucial role of SLIM1 in sulfur deficiency to coordinate sulfur availability with sugar metabolism.

## Materials and methods

### Plant material

Arabidopsis Columbia ecotype (Col-0) was used as the parental line for the *slim1* deletion line (*slim1_KO*). Deletion in the SLIM1 gene was generated using the CRISPR/Cas9 method (Fauser *et al.*, 2014). Using a CRISPR-P 2.0 online tool (H. Liu *et al.*, 2017), two sgRNA were designed that targeted exons 1 and 2 of the *SLIM1* locus in Arabidopsis (SLIMsgRNA1: CTATGTCCGTAGCAGACATC and SLIMsgRNA2: GGGGTAATAACGCTGACAGG), and were assembled in a modified pEn-Chimera. The sgRNAs were then combined with Cas9 nickase by Gateway® cloning into the pDe-Cas9 binary plasmid. The construct was transformed into Arabidopsis by *Agrobacterium tumefaciens*-mediated transformation (Clough and Bent, 1998). The construct for SLIM1 expression was created by cloning the SLIM1 coding regions under the control of its native promoter (2088 bp upstream ATG codon) into vector pGWB419 (Nakagawa *et al.*, 2007) using the primers listed in Supplementary Table S1. The binary plasmid was transferred to *Agrobacterium tumefaciens* GV3101 and introduced into the *slim1_KO* mutant with the floral dip method (Clough and Bent, 1998). *gin2/hxk1* mutant and *HXK_OE3.2* overexpressor together with their parental ecotypes Landsberg erecta (Ler) and Bensheim, respectively, were a kind gift of Prof. Leszek Kleczkowski.

### Plant growth media and conditions

**Fig. 1.**
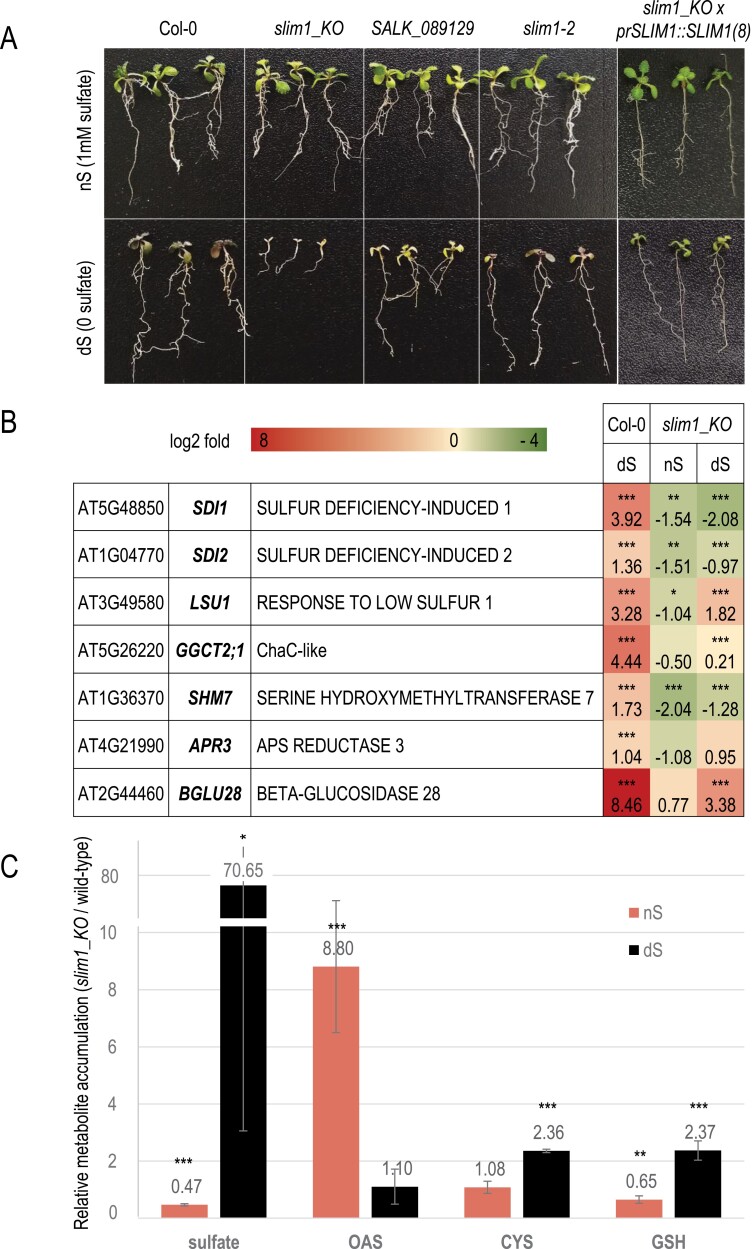
The *slim1* mutant shows a disturbed response to sulfur deficiency. (A) Growth phenotype of a newly constructed *slim1_KO* and other described *slim1* mutants after 12 d in normal sulfate supply (nS) and no sulfate (dS) conditions. *slim1_KO* × *prSLIM1::SLIM1(8)* line is a *slim1_KO* mutant transformed with *SLIM1* coding sequence under its native promoter. (B) Heatmap showing the log2-fold change of expression of ‘OAS cluster’ and *BGLU28* genes in 7-day-old seedlings transferred to either nS or dS medium for 48 h (where expression of the wild-type in nS is 1). Asterisks indicate statistical difference either between expression level in nS versus dS (for the wild-type (Col-0)) or between Col-0 and the *slim1_KO* mutant in the same treatment (Student’s *t*-test; *P≤0.05, **P≤0.01, ***P≤0.001). The experiment was repeated three times. (C) Relative sulfur metabolite contents of the shoots of *slim1_KO* mutant grown for 14 d in nS and dS conditions (where the content of a given metabolite in the wild-type in the same condition is 1). GSH: reduced glutathione; OAS: *O*-acetylserine. Asterisks indicate statistical difference between Col-0 and the *slim1_KO* mutant in the same treatment (Student’s *t*-test; *P≤0.05, **P≤0.01, ***P≤0.001). The experiment was repeated four times. The absolute values of the metabolite contents are in Supplementary Table S3.

Arabidopsis seeds were surface-sterilized using a vapor-phase seed sterilization method (Zientara *et al.*, 2009), exposed to a temperature of 4 °C in the dark for 3 d, and then germinated on 0.7% agar containing half-strength Hoagland’s medium (Schat *et al.*, 1996). For sulfur starvation experiments, two types of medium were used in the experiments that contained either a normal sulfur (‘nS’; 1 mM sulfate) or sulfur deficient supply (‘dS’; 10 μM sulfate). In some cases, medium with no sulfate was used (results presented in Fig. 1A). Seedlings were grown in a climate chamber at 23 °C with a 16/8 h light/dark photoperiod for the indicated amount of time. The plates were maintained vertically to allow gravitational elongation of the root apex. For the glucose sensitivity/germination assay, glucose or mannose was added to the medium at the indicated concentration. For gene expression studies seedlings were grown for 7 d in liquid medium in 12-wells plates, dried on a paper towel and transferred to the same medium but containing 5% glucose or 5% mannitol where they were grown for 17 h before harvesting. In another experimental set-up, for short-term sulfur starvation, 7-day-old seedlings grown on either 1% or 3% glucose were transferred to either nS or dS medium and an adequate glucose concentration, and the material was collected after 48 h. For the measurements of sulfur metabolites, seedlings were grown for 14 d in either nS or sulfur deficiency dS medium and only shoots were collected.

### Measurement of anthocyanin

Extraction and photometric determination of anthocyanin content in seedlings were performed according to the following protocol. Pools of seedlings were weighed, frozen in liquid nitrogen, and homogenized with a mini pestle. To the homogenized plant material, 600 µl extraction buffer (acidic methanol, 1% (v/v) HCl) was added and the samples were incubated overnight with moderate rotation at 4 °C in the dark. Next, 400 µl of deionized water was added, followed by chlorophyll extraction with 400 µl chloroform. The homogenate was cleared by centrifugation at 13 400 *g* for 2 min and the supernatant was used for photometric measurements. To quantify anthocyanin in the samples the following equation was used: (*A*_530_ – 0.25*A*_657_)/*M* (g) = relative units of anthocyanin (where *A* is absorption and *M* is plant fresh weight) (Rabino and Mancinelli, 1986).

### Yeast one-hybrid assay

All enzymes used for cloning the constructs used in this study were obtained from Thermo Fisher Scientific, Lithuania. To construct the reporter plasmid for a yeast one-hybrid (Y1H) assay, a 958 bp DNA fragment harboring sequence upstream *PAP1* open reading frame was ligated into the *Sac*I and *Mlu*I sites of the pHIS2.1 vector (Takara Bio USA, Inc.), upstream of the *HIS3* minimal promoter. Two short DNA sequences present in the *PAP1* promoter and matching *cis* binding sites for EIN3 or SLIM1 (Wawrzynska *et al.*, 2010; Meng *et al.*, 2020) were PCR amplified with the pPAP_UPE and HISR or pPAP_EIN and HISR primers, respectively (Supplementary Table S1) flanking 1437 bp of the pHIS2.1 vector. The PCR products were then inserted into the *Mlu*I*/Bam*HI sites of the pHIS2.1 vector, and the corresponding constructs were named pUPE-HIS and pEIN-HIS, respectively. The open reading frame of SLIM1 or EIN3 was fused in-frame with the GAL4 activation domain in a pDEST-GADT7 vector (Rossignol *et al.*, 2007) to generate the effector plasmid (SLIM1/EIN3 × pGAD). Pairs of these reporter and effector plasmids were introduced into yeast strain Y187 (Takara Bio USA) and the transformants were selected on SD medium lacking leucine and tryptophan. Transformed colonies were then spotted on SD/–His/–Leu/–Trp medium with different concentrations of 3-aminotriazole and cultured at 28 °C for 3 d. All plasmids used in the assay are described in Supplementary Table S1.

### Gene expression analysis

Total RNA was isolated from seedlings using TRI Reagent (Molecular Research Center, Cincinnati, OH, USA) according to the manufacturer’s protocol (Chomczynski and Sacchi, 1987). For quantitative real-time PCR (qRT-PCR) analysis, 2 µg RNA was reverse-transcribed using a Maxima First Strand cDNA Synthesis Kit (Thermo Fisher Scientific, Lithuania) according to the manufacturer’s instructions. The 100-fold dilution of synthesized cDNA was used as a template for qRT-PCR. The qRT-PCR reaction was performed using a PikoReal Real-Time PCR System (Thermo Fisher Scientific). The *TUBULIN 3* gene (*TUA3*; At5g19770) was selected as an internal control to normalize the quantity of total RNA present in each sample. Stable expression of *TUA3* under all experimental conditions was confirmed by its steady *C*_q_ values in qRT-PCR (*C*_q_: 20.52 ± 0.57; mean ±SD, n=180) (Supplementary Fig. S1A) but also by comparing its expression to two other reference genes *ACTIN2* (*ACT2*, At3g18780) (Guenin *et al.*, 2009) and *TIP41* (At4g34270) (Czechowski *et al.*, 2005) (Supplementary Fig. S1B). All primers used for qRT-PCR are listed in Supplementary Table S2. The specificity of the forward and reverse primers for the candidate gene was checked using the NCBI-BLAST website (http://www.ncbi.nlm.nih.gov/blast/Blast.cgi, RRID:SCR_004870) and melting curve analysis following qRT-PCR. The reaction mixture (6 µl) contained 3 µl of DyNAmo Color Flash SYBR Green Master Mix (Thermo Fisher Scientific), 0.6 µM each of forward and reverse primers, and 1 µl of cDNA template. qRT-PCR was carried out in triplicate (technical repeats) of three to four biological repeats to ensure the reproducibility of the results. Relative gene expression levels were calculated using the ΔΔ*C*_t_ method (Livak and Schmittgen, 2001).

### Metabolite analysis

Shoots were harvested and frozen in liquid nitrogen before the extraction of metabolites. Sulfate content was determined by high-performance anion-exchange chromatography with conductivity detection using a Dionex ICS-2000 system as described (Hubberten *et al.*, 2012). *O*-acetylserine (OAS) levels were determined after derivatization using an amino acid analysis protocol (Hubberten *et al.*, 2012) and analysed by the Dionex Summit HPLC system. Cysteine and glutathione were extracted using a protocol described (Hubberten *et al.*, 2012). The labeled products were analysed by HPLC using a LiChrospher 60 RP-select B (5 μm) LiChroCART 125-4 chromatography column (Merck, Germany) in a Dionex Summit HPLC system.

### β-Glucuronidase activity assays

Qualitative and quantitative assays of β-glucuronidase (GUS) activity were performed as described (Wawrzynska *et al.*, 2010). Leaves of *Nicotiana benthamiana* were infiltrated with *Agrobacterium tumefaciens* GV3101 strain harboring both reporter (i.e. 958 bp DNA fragment upstream *PAP1* start codon ligated into the *Sac*I and *Not*I sites of the pGG vector controlling *uidA* gene; Wawrzynska *et al.*, 2010) and effector (i.e. SLIM1 or EIN3) encoding plasmids based on pGWB420 (Nakagawa *et al.*, 2007). The mutations of the SLIM1/EIN3 binding sites in the promoter region of *PAP1* (*promPAP1m1* and *promPAP1m2*, respectively) have been introduced by PCR with mutated primers. To ensure an equal physiological state, all leaves were infiltrated with a control (reporter plasmid alone; left side of the leaf) and the tested interaction (right side of the leaf). Three to four discs of 10 mm diameter were cut from the infiltrated leaf and used directly for staining or frozen and later used for a quantitative assay. All oligonucleotides and plasmids used in the assay are described in Supplementary Table S1.

### Statistical analyses

The statistical difference between the two genotypes (*slim1_KO* versus Col-0) was tested by a two-tailed, unpaired Student’s *t*-test in Microsoft Excel. The statistical significance of group differences was assessed with Fisher’s least significant difference (LSD) test of two-way ANOVA using Statistica 12 software (StatSoft Polska Sp. z o.o., Poland). Differences were considered significant at a *P*-value <0.05. Asterisks indicate significant differences between values of two genotypes within one treatment (*P<0.05, **P<0.01, and ***P<0.001). All experiments were independently repeated at least three times.

## Results

### The *slim1_KO* mutant demonstrates a more severe phenotype than other *slim1* mutants in sulfur deficiency

Several *slim1* mutants described in the literature show different phenotypes in sulfur-deficient conditions. In the pioneering work identifying SLIM1 as a master regulator of transcription during sulfur deficiency, two point mutants, *slim1-1* and *slim1-2*, obtained after ethyl methanesulfonate treatment, exhibited a 30% reduction of root growth compared with the parental line (Maruyama-Nakashita *et al.*, 2006). The only available *slim1* T-DNA line with the disruption of the 5ʹ-untranslated region (UTR) (SALK_089129) was recently compared with those original *slim1* mutants and displayed similar defects in the transcriptional response to sulfur deficiency, although its phenotype was not described (Dietzen *et al.*, 2020). Here, we constructed and characterized the *SLIM1* deletion mutant that was generated by applying CRISPR/Cas9 technology. The genomic region covering the deletion was amplified and sequenced. The deletion starts from the 10th residue of the SLIM1 protein with the open reading frame reconstituted from residue 310 up to 567 (stop codon) (Supplementary Fig. S2). We presumed that such a chimeric protein cannot function as a transcription factor since it is missing the DNA binding domains, and that therefore it is a knock-out mutant. To check this hypothesis, we cloned a truncated *SLIM1* coding sequence from *slim1_KO* to check its ability to activate reporter gene expression in a Y1H assay using a construct containing the SLIM1 binding site (*UPE-box*) (Wawrzynska *et al.*, 2010). The truncated SLIM1 was unable to induce *HIS3* gene expression thus preventing growth of the yeast on selective media (Supplementary Fig. S3A). However, when such truncated protein was fused with the GAL4 DNA binding domain and used in a yeast two-hybrid (Y2H) assay it was able to autoactivate the reporter gene and enable yeast growth on selective media, thus proving the presence of the activation domain in the chimeric SLIM1 protein expressed in the *slim1_KO* mutant (Supplementary Fig. S3B). Additionally, we checked for any off-target effects of the CRISPR/Cas9-construct on the coding sequences of the closest homologs of *SLIM1*, known to take part in sulfur deficiency response, namely *EIN3* and *EIL1* (Wawrzynska and Sirko, 2016; Dietzen *et al.*, 2020). Neither *EIN3* nor *EIL3* coding sequences had mutations (data not shown).

The *slim1_KO* mutant displays a clear phenotype when grown in sulfur-deficient conditions, which is even more severe than that observed in SALK_089129 and *slim1-2* (Fig. 1A). This phenotype can be fully rescued by the re-introduction of the *SLIM1* coding sequence under the control of its native promoter to the *slim1_KO* mutant (Fig. 1A; Supplementary Fig. S4). Next, we assayed the transcriptional response of the *slim1_KO* mutant when exposed to short 2-day sulfur starvation. We followed the expression of the ‘OAS cluster’ genes and *β-GLUCUSIDASE 28* (*BGLU28*), which are known to be mostly dependent on *SLIM1* transcriptional activity (Maruyama-Nakashita *et al.*, 2006; Hubberten *et al.*, 2012). In contrast to the wild-type, none of these genes demonstrated induction (or were less induced) by sulfur deficiency (Fig. 1B), providing evidence that the truncated version of *SLIM1* expressed in the *slim1_KO* mutant is non-functional. Additionally, we measured the content of several metabolites connected with sulfur metabolism, namely sulfate, OAS, cysteine, and glutathione (Fig. 1C). We chose to assay just shoots because previous data for other *slim1* mutants suggested that this is where changes in most sulfur metabolites are noticed (Maruyama-Nakashita *et al.*, 2006; Dietzen *et al.*, 2020; Yamaguchi *et al.*, 2020; Jobe *et al.*, 2021). Surprisingly, upon normal sulfur supply, the *slim1_KO* mutant accumulates almost nine times more OAS than wild-type plants but significantly less sulfate and glutathione. However, after 14 d of growth in sulfate-deficient conditions, the mutant accumulated 70 times more sulfate and produced more than twice as much cysteine and glutathione in shoots compared with the wild-type (Fig. 1C; Supplementary Table S3).

### The *slim1_KO* mutant is hypersensitive to glucose

**Fig. 2.**
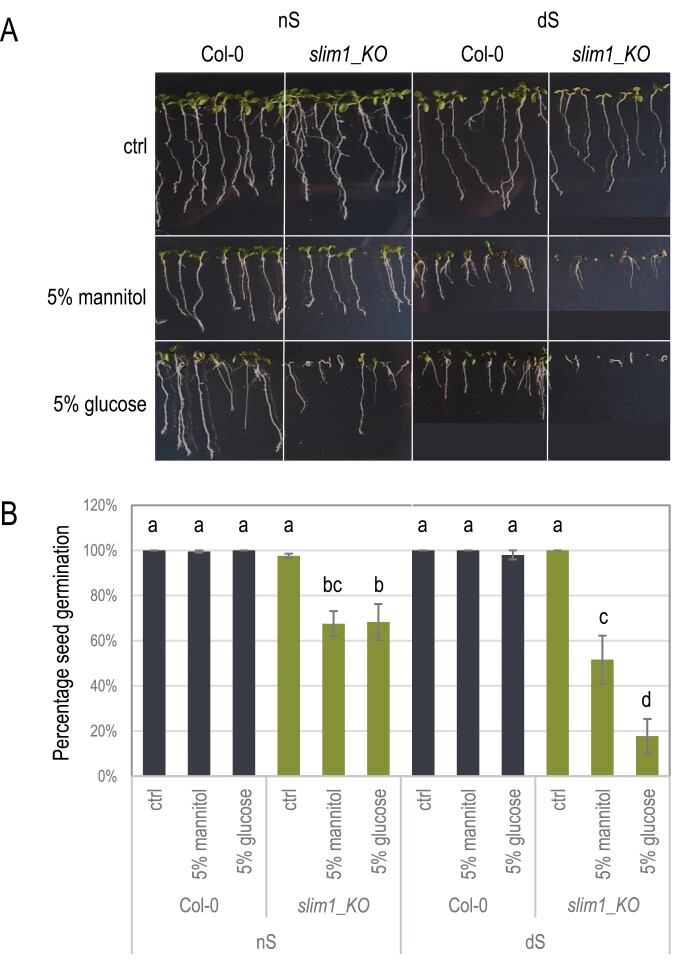
The *slim1_KO* mutant is sensitive to high glucose. (A) Growth phenotype of seedlings grown 12 d in the presence of either 5% glucose or 5% mannitol (osmotic control) and different sulfate availability (normal sulfate supply (nS) and sulfate deficiency (dS)). (B) Mean percentage *g*ermination rate with standard errors of Col-0 and the *slim1_KO* mutant in the presence of either 5% glucose or 5% mannitol at 5 d post-sowing. Different lowercase letters indicate statistically significant differences between the treatments (two-way ANOVA, Fisher’s LSD test, *P*≤0.05). The experiment was repeated six times with different sets of *slim1_KO* mutant seeds collected simultaneously with the wild-type seeds.

Elevated glucose levels induce a developmental arrest of light-grown Arabidopsis seedlings, which enables the investigation of the glucose signaling pathway in higher plants (Zhou *et al.*, 1998). Germination as well as greening and expansion of cotyledons are completely suppressed when the glucose level in the standard culture medium is raised to 6%, in contrast to the growth-promoting 2%. Many of the glucose-sensitive, as well as glucose-insensitive, mutants, have defects in the ethylene signaling pathway, providing evidence that glucose signaling is tightly linked to this pathway and that both pathways have opposite roles in the germination process (Zhou *et al.*, 1998). Most importantly to our studies, the *ein3* mutant appears to be sensitive to glucose, while overexpression of *EIN3* in Arabidopsis significantly decreases glucose sensitivity (Yanagisawa *et al.*, 2003). Since EIN3 is a close homolog of SLIM1, we hypothesized that the transcriptional regulator of sulfur deficiency, SLIM1, might also play a role in glucose signaling. We performed root growth assays to test the *slim1_KO* mutant’s sensitivity to glucose in sulfur-sufficient (nS) and sulfur-deficient (dS) conditions (Fig. 2A). While the growth of the wild-type seedlings was similarly inhibited by osmotic stress control (5% mannitol) and 5% glucose, irrespective of sulfate availability in the growth media, the *slim_KO* mutant exhibited a severe growth reduction phenotype in the combined presence of 5% glucose and sulfur limitation. Similar phenotypes were also observed for other *slim1* mutants (Supplementary Fig. S5). During these tests, we also noticed that the *slim1_KO* mutant had germination problems, especially when grown under osmotic pressure and sulfur limitation conditions. Therefore, we separately checked the germination ability of the *slim1_KO* mutant (Fig. 2B). All seeds of the wild-type plants germinated irrespective of the applied conditions, while the *slim1_KO* mutant again displayed the most severe phenotype (less than 20% seed germination) when plated on medium with 5% glucose and no sulfate. The *slim1_KO* mutant also showed sensitivity to osmotic stress, as it had a lower germination rate in the presence of 5% mannitol with no sulfate limitation (Fig. 2B). All of these results support the idea that SLIM1 mediates responses not only to restricted sulfur availability but also to glucose and osmotic stress.

### ‘OAS cluster’ genes are SLIM1-dependent but also respond to glucose availability

**Fig. 3.**
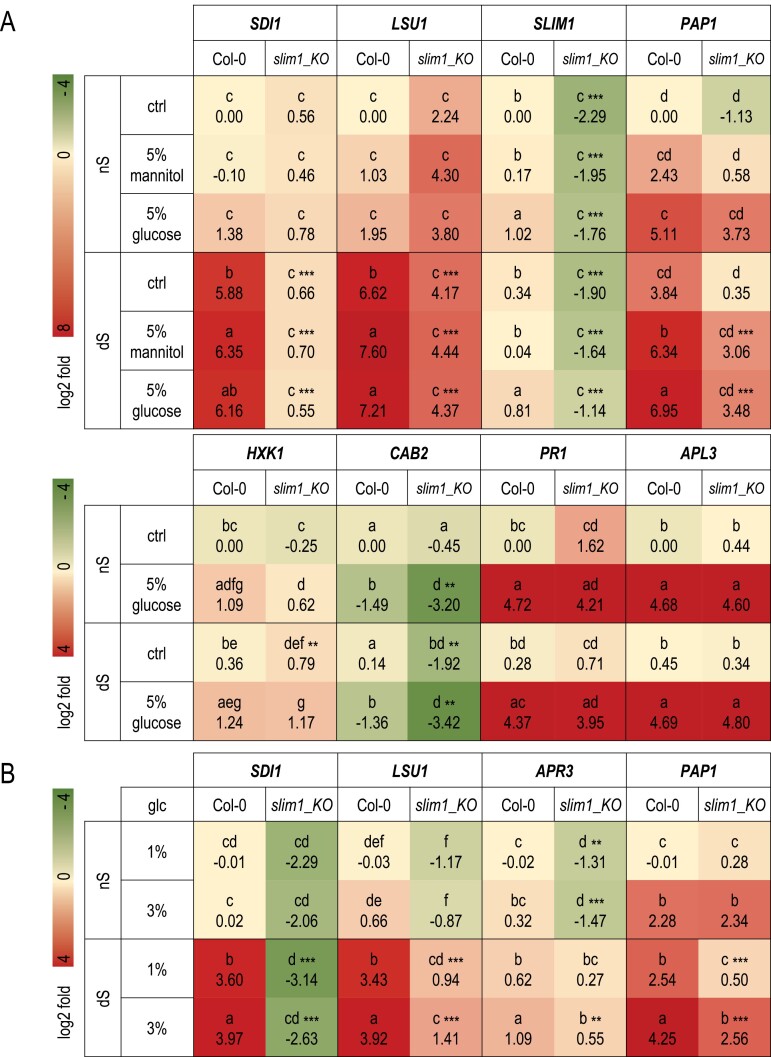
SLIM1 enhances the expression of selected genes in high glucose but only after short-term sulfur starvation. (A) Heatmap showing the log2-fold change of expression of selected genes in 7-day-old seedlings grown in either normal sulfate supply (nS) or sulfate deficiency (dS) conditions and transferred for 17 h to either 5% glucose or 5% mannitol. (B) Heatmap showing the log2-fold change of expression of selected genes in 7-day-old seedlings grown in either 1% or 3% glucose (nS) and transferred for 48 h to either nS or dS conditions maintaining a consistent glucose concentration. Different lowercase letters indicate significant differences between the different treatments (two-way ANOVA with ‘genotype’ and ‘treatment’ as fixed factors followed by Fisher LSD post hoc test, P≤0.05). Asterisks next to letters mark a highly significant difference between genotypes in the same treatment (**P≤0.01, ***P≤0.001). The experiment was repeated four times.

Next, we checked the transcriptional response to high glucose of the selected genes known to respond to sulfur deficiency in a SLIM1-dependent manner, namely *SULFUR DEFICIENCY-INDUCED 1* (*SDI1*) and *RESPONSE TO LOW SULFUR 1* (*LSU1*) (Wawrzynska and Sirko, 2016; Rakpenthai *et al.*, 2022). Both genes belong to so-called ‘OAS cluster’ genes as their expression follows *O*-acetylserine (OAS; cysteine backbone) diurnal fluctuations, but they are also on the list of genes that are regulated by SLIM1 (Maruyama-Nakashita *et al.*, 2006; Hubberten *et al.*, 2012). *SDI1* encodes a major repressor controlling the biosynthesis of glucosinolates under sulfur deficiency conditions while the precise role of LSU1 in plant metabolism is not yet established (Aarabi *et al.*, 2016, 2020; Niemiro *et al.*, 2020). Seven-day-old seedlings grown on either nS or dS medium were treated for 17 h with 5% glucose or 5% mannitol (osmotic control), and gene expression was assayed (Fig. 3A). Both *SDI1* and *LSU1* demonstrated huge overexpression in sulfur-deficient conditions, with the level of expression even more heightened by the presence of osmotic agents. During normal sulfur supply, the presence of glucose seemed to have a positive effect on *SDI1* and *LSU1* expression, although it was not statistically significant in the conditions of our experiment. This positive effect was visible in both the wild-type and *slim1_KO* mutant, suggesting that it is SLIM1 independent (Fig. 3A). Additionally, we also determined whether the expression of *SLIM1* was affected by sulfur deficiency or glucose treatment and assayed the level of the *PAP1* transcript as it is involved in glucose signaling and transcriptionally negatively controlled by EIN3 (Meng *et al.*, 2020). Surprisingly, *SLIM1* expression was increased by glucose treatment, but not affected by the sulfur status of the environment (Fig. 3A). In agreement with literature data (Pourtau *et al.*, 2006) a strong activation of *PAP1* expression by mannitol and even stronger by glucose irrespective of sulfur availability was noticed. Interestingly, *PAP1* was also induced by sulfur starvation and this effect was totally abolished in the *slim1_KO* mutant (Fig. 3A).

Next, we evaluated the expression level of *HXK1*. In wild-type plants, *HXK1* was slightly induced by osmotic stress in both nS and dS conditions. Although an increase in the *HXK1* transcript level in the above conditions could also be noticed in the *slim1_KO* mutant, we observed its unexpected diminution in nS conditions (Fig. 3A). To further elucidate the connection between SLIM1 and HXK1 we explored the expression of glucose-responsive genes from three distinct pathways: (i) a HXK1-dependent pathway (*CHLOROPHYLL A/B-BINDING PROTEIN 2* (*CAB2*)), (ii) a HXK1 enzymatic activity-dependent pathway (*PATHOGENESIS-RELATED GENE 1* (*PR1*), induced by unknown metabolite(s) downstream in the glycolytic pathway), and (iii) a HXK1-independent pathway (gene encoding the large subunit of ADP-Glucose Pyrophosphorylase (*APL3*) involved in starch biosynthesis) (Xiao *et al.*, 2000). While both *PR1* and *APL3* similarly responded to glucose in the wild-type and *slim1_KO* mutant irrespective of the sulfur status in the environment, surprisingly, *CAB2* expression was affected in the *slim1_KO* mutant. SLIM1 seems to positively participate in signaling the glucose status via HXK1, which is especially noticeable during dS conditions (Fig. 3A). The transcription of *CAB2* is diminished in the *slim1_KO* mutant even without glucose addition and it is even more repressed by glucose than the one observed for the wild-type under similar conditions (Fig. 3A).

In another experimental set-up, we decided to check the impact of short-term (2 d) sulfur starvation on the expression of genes in 7-day-old seedlings grown in the presence of high glucose (3%) (Fig. 3B). *PAP1* was induced by glucose and sulfur starvation in the wild-type while only glucose promoted its expression in the *slim1_KO* mutant. Elevated glucose was also beneficial for the expression of *SDI1* and *LSU1* in dS conditions, as a significant increase was observed in addition to the induction by dS. In this experiment, we additionally assayed the expression of *APR3*, which is another gene belonging to the ‘OAS cluster’ but also known to respond positively to glucose (Hesse *et al.*, 2003; Hubberten *et al.*, 2012). As expected, its pattern of expression was similar to *SDI1* and *LSU1* (Fig. 3B).

### Hexokinase 1 participates in the induction of the ‘OAS cluster’ in S deficiency

**Fig. 4.**
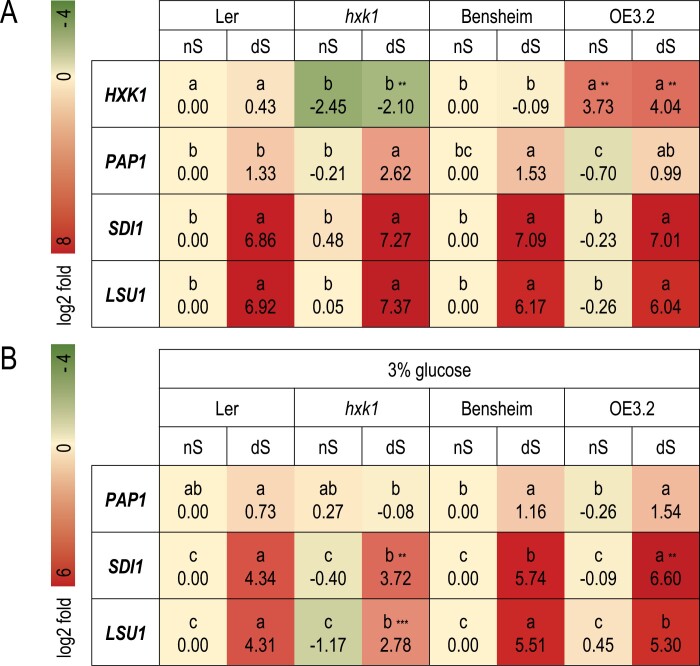
Hexokinase 1 participates in mediating SLIM1-dependent transcriptional response but only after short-term sulfur starvation. (A) Heatmap showing the log2-fold change of expression of selected genes in 7-day-old seedlings grown in either normal sulfate supply (nS) or sulfate deficiency (dS) conditions. (B) Heatmap showing the log2-fold change of expression of selected genes in 7-day-old seedlings grown in nS in the presence of 3% glucose and transferred for 48 h to either nS or dS conditions maintaining a consistent glucose concentration. Different lowercase letters indicate significant differences between the different treatments (two-way ANOVA with ‘genotype’ and ‘treatment’ as fixed factors followed by Fisher’s LSD *post hoc* test, *P*≤0.05). Asterisks next to letters mark a highly significant difference between genotypes in the same treatment (**P≤0.01, ***P≤0.001). The experiment was repeated three times.

Because we noticed the positive impact of glucose on the expression of ‘OAS cluster’ genes and changes in *HXK1* expression in the *slim1_KO* mutant, the role of HXK1 in this process was assayed. The changes induced by glucose can be eliminated or exaggerated by decreasing or increasing the amount of the glucose sensor HXK in transgenic Arabidopsis plants (Jang *et al.*, 1997). Furthermore, this effect is uncoupled from glucose metabolism and depends solely on glucose signaling events (Jang *et al.*, 1997). To determine if HXK1 may have any effect on ‘OAS cluster’ gene expression under sulfur deficiency conditions, we grew Arabidopsis lines with altered *HXK1* expression in nS and dS conditions for 7 d. Because these mutants have different genetic backgrounds, the respective controls were grown simultaneously—Landsberg erecta for the *hxk1* null mutant and Bensheim for the *HXK1* overexpressor line OE3.2. The expression of *SDI1* and *LSU1* was similarly induced by dS conditions in all tested lines (Fig. 4A). Interestingly, *PAP1* expression was also induced by dS in all tested mutants, but to a significantly higher level in the *hxk1* mutant. This might suggest a negative control of HXK1 over the sulfur deficiency-dependent increase in *PAP1* mRNA.

In a separate experiment, we investigated the effects of short-term (2 d) sulfur deficiency on gene expression in 7-day-old seedlings grown in the presence of high glucose (3%) (Fig. 4B), similar to the experimental set-up described in Fig. 3B and using the same set of plant lines as in Fig. 4B. Arabidopsis plants with altered *HXK1* expression showed significant changes in the glucose-dependent increase in the level of *SDI1* and *LSU1* in comparison with adequate genetic backgrounds, implying that HXK1 might be involved in sensing/transmitting the sugar signal to ‘OAS cluster’ genes. Both *SDI1* and *LSU1* expression was increased to a lower level in the *hxk1* mutant, while in the *HXK1* overexpressor it was higher for *SDI1. LSU1* induction by dS conditions reached a similar level in the *HXK1* overexpressor and wild-type. The results suggest a positive role for HXK1 in SLIM1-dependent transcriptional induction. In this experimental set-up, we did not observe a significant increase of the *PAP1* transcript in dS in *hxk1* mutant, although a positive impact of the lack of sulfur on *PAP1* expression in all other genetic backgrounds could be noticed. This might imply that HXK1 is involved in the increase of *PAP1* transcription under dS conditions.

### *PAP1* expression and anthocyanin content are SLIM1 dependent during S deficiency

**Fig. 5.**
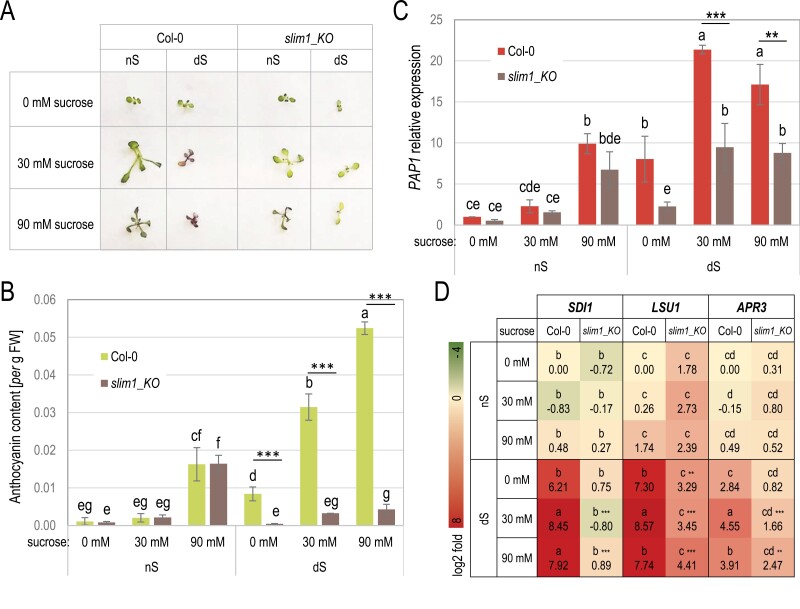
The *slim1_KO* mutant does not produce anthocyanin under sulfur-deficiency stress. (A) Growth phenotype of seedlings grown 12 d in the presence of increased sucrose concentration and different sulfate availability (normal sulfate supply (nS) and sulfate deficiency (dS)). (B) Anthocyanin content in 12-day-old seedlings grown in the presence of increased sucrose concentration and different sulfate availability. (C) Relative *PAP1* expression in 12-day-old seedlings grown in the presence of increased sucrose concentration and different sulfate availability. (D) Heatmap showing the log2-fold change of expression of selected ‘OAS cluster’ genes in 12-day-old seedlings grown in the presence of increased sucrose concentration and different sulfate availability. Different lowercase letters indicate significant differences between the different treatments (two-way ANOVA with ‘genotype’ and ‘treatment’ as fixed factors followed by Fisher’s LSD *post hoc* test, *P*≤0.05). Asterisks next to letters mark a highly significant difference between genotypes in the same treatment (**P≤0.01, ***P≤0.001). The experiment was repeated three times.

As previously observed (Figs 3, 4) the expression of *PAP1* is induced by sulfur deficiency, and this effect seems to be dependent on SLIM1. Because the *PAP1* gene was previously described as sucrose dependent in the process of anthocyanin biosynthesis induction in Arabidopsis (Hesse *et al.*, 2003; Teng *et al.*, 2005), we decided to assay the anthocyanin content in plants grown for 7 d in nS and dS conditions accompanied by increased sucrose concentrations (Fig. 5B). The *slim1_KO* mutant seedlings differed phenotypically from the wild-type but only in dS conditions (Fig. 5A). Indeed, in nS conditions, the anthocyanin level grew in direct proportion to the level of sucrose in the medium and similarly in the wild-type and the *slim1_KO* mutant. However, in dS conditions, the content of anthocyanin in the wild-type was already high under no sucrose conditions and grew further together with increasing sucrose concentrations, while this effect was apparently absent in the *slim1_KO* mutant (Fig. 5B). This suggests that anthocyanin synthesis under dS conditions is controlled by SLIM1. The induction of *PAP1* accompanied by increased anthocyanin levels in the wild-type under dS conditions, despite the absence of external sucrose, could be explained by the fact that the internal sucrose content increased during sulfur deficiency (Bielecka *et al.*, 2015). Next, we determined the expression level of selected genes in the same material. As concluded from the anthocyanin level, the level of *PAP1* expression was positively correlated with these data, again confirming that SLIM1 is involved in the control of *PAP1* activation under sulfur deficiency (Fig. 5C). Interestingly, though the level of *PAP1* transcript was not increased by 90 mM as compared with 30 mM sucrose, the level of anthocyanins built up further. This can be explained by the contribution of other factors that might be induced by dS and sucrose thus accelerating the accumulation of anthocyanins. The anthocyanin synthesis pathway is regulated by a suite of transcription factors that often form complexes affecting their transcriptional activity at the post-translational level (Cappellini *et al.*, 2021). We also assayed the level of transcription of three genes belonging to the ‘OAS cluster’ (Fig. 5D). *SDI1*, *LSU1*, and *APR3* showed increased levels of mRNA in dS and the addition of 30 mM sucrose had an additional positive effect on their transcription. However, this effect was not further enhanced with higher sucrose concentration, suggesting that there is a certain threshold of mRNA levels of these three genes. The presence of sucrose in the medium did not affect the expression of *SDI1*, *LSU1*, and *APR3* in nS conditions. Interestingly, out of these three genes, only *LSU1* demonstrated increased transcript levels in nS in the absence of SLIM1 and this was also noticed in seedlings grown in the glucose treatment experiment (Figs 3A, 5D). Similar experiments to that presented in Fig. 5 were performed for the other *slim1* mutants with comparable results (Supplementary Fig. S6). However, the effects noticed for *slim1_KO* mutant were milder in *slim1-2* and SALK_089129 lines.

## SLIM1 can bind and activate the *PAP1* promoter

**Fig. 6.**
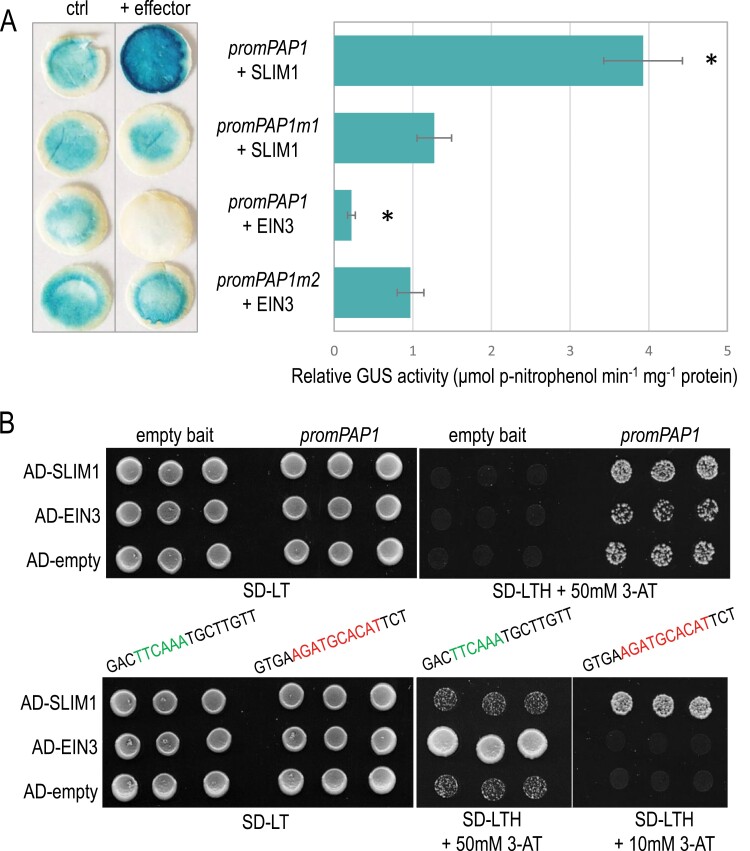
SLIM1 binds to the *PAP1* promoter and activates *PAP1* expression. (A) Histochemical GUS assay and quantitative measurements of GUS activity in transiently transformed *Nicotiana benthamiana* at 3 d after infiltration with the reporter alone (ctrl) (right side of the leaf) or in combination with the effectors (EIN3 or SLIM1, left side of the same leaf). The *uidA* gene in the reporter constructs was driven by the DNA fragment 958 nt upstream of the ATG codon of the *PAP1* gene. *promPAP1m1* and *promPAP1m2* are the *PAP1* promoters with the mutated biding sites for SLIM1 and EIN3, respectively, which are shown in the lower panel of (B). The relative GUS activity was calculated by dividing the GUS activity observed for the right side of the leaf by the activity of the corresponding left side of the leaf (ctrl). Error bars represent the SD of mean GUS activity values derived from three independent isolations. Asterisks indicate statistically significant differences (Student’s *t*-test, *P*≤0.05) as compared with the corresponding controls without effectors. (B) Yeast one-hybrid screening. In the upper panel growth of yeast harboring *HIS3* reporter fused to DNA fragment 958 nt upstream of the ATG codon of the *PAP1* gene cotransformed with either the EIN3 or SLIM1 effector. In the lower panel, the *HIS3* reporter was fused to DNA fragments from the sequence of the *PAP1* promoter framing the binding sites for EIN3 (in green) and SLIM1 (in red) (Wawrzynska *et al.*, 2010; Meng *et al.*, 2020) and cotransformed with either the EIN3 or SLIM1 effector. Yeasts were grown for 3 d at 30 °C on a selective medium lacking leucine, tryptophan, and histidine with either 10 mM or 50 mM 3-aminotriazole and photographed. On the left-hand side, yeast growth on the control medium lacking leucine and tryptophan is shown. Three independently transformed yeast colonies are shown.

Our findings suggest that SLIM1 directly and positively regulates *PAP1* expression during sulfur deficiency. Therefore, we decided to determine if SLIM1 is able to bind to the *PAP1* promoter. We identified a putative SLIM1 binding site core (AGATGCACAT) within the promoter of *PAP1*, which is only 21 nucleotides downstream of the published EIN3 binding site (Meng *et al.*, 2020). To confirm the binding of SLIM1 to the *PAP1* promoter, we performed a transactivation experiment in *Nicotiana benthamiana.* Transient expression data showed that co-expression of SLIM1 increased the expression of a *promoterPAP1*::*GUS* reporter gene (containing 958 bp upstream PAP1 start codon) (Fig. 6A), indicating that SLIM1 directly binds to the *PAP1* gene promoter to activate its expression. EIN3, which was also used in these studies, negatively regulated the same reporter construct, which is in agreement with the literature (Meng *et al.*, 2020). To additionally confirm SLIM1 binding to the predicted binding site, we decided to mutagenize it (*promPAP1m1*) and check the GUS expression. Co-expression with SLIM1 showed GUS activity at the level of control, i.e. unmutated *promoterPAP1*::*GUS* reporter gene expressed with no effector (Fig. 6A). Alongside the above mutation, we also mutagenized the EIN3 binding site (*promPAP1m2*). As expected, no visible differences in GUS activity were noticed between *promPAP1m2* co-expressed with EIN3 and control, i.e. unmutated *promoterPAP1*::*GUS* reporter gene expressed with no effector (Fig. 6A).

To confirm these data by an independent assay, we performed a Y1H test. However, the DNA fragment of the *PAP1* promoter (958 bp upstream of the start codon) appeared to be able to autoactivate the reporter gene, probably due to the interaction with yeast transcription factor(s) (Fig. 6B). Therefore, we decided to clone only the short DNA regions (17 bp) framing the binding sites for EIN3 and SLIM1 (Wawrzynska *et al.*, 2010; Meng *et al.*, 2020). The yeast strain containing only the constructs harboring the indicated fragments of the *PAP1* promoter failed to grow on a selective medium. In contrast, yeast strains containing both the fragments of the *PAP1* promoter and SLIM1 or EIN3 expression plasmids grew on the selective medium. Interestingly, EIN3 failed to bind to the selected SLIM1 binding site, while SLIM1 was unable to recognize and bind to the EIN3 binding site (Fig. 6B). This underlines the fact that both transcription factors have different and specific binding sites in the *PAP1* promoter.

## Discussion

SLIM1 has been described in Arabidopsis as a crucial transcription factor governing the expression of dozens of genes during sulfur deficiency. Moreover, its role in cadmium and arsenic resistance and signaling has recently been underlined (Jobe *et al.*, 2021; Yamaguchi *et al.*, 2020). Here, we demonstrated that SLIM1 is an important player in the process of coordination of sugar metabolism and signaling with sulfur availability.

We describe here the construction of a new *slim1_KO* mutant using CRISPR/Cas9 technology. Similar experiments to those presented in Fig. 5 were performed for previously described *slim1* mutants with comparable results (Supplementary Fig. S6). However, the effects observed for the *slim1_KO* mutant were stronger than for *slim1-2* and SALK_089129 lines. All the phenotypic observations, presented in Fig. 1 and Supplementary Figs S5, S6 together with the metabolite data presented in Fig. 1C, led us to believe that both *slim1-2* and SALK_089129 mutants can still accumulate SLIM1 proteins although not fully functional. The *slim1-2* point mutation in the DNA-binding domain might affect the ability of SLIM1 to bind to the *cis*-acting regulatory element (*UPE-box*) of targeted genes, while the SALK_089129 T-DNA insertion in the 5ʹ-UTR of the SLIM1 coding sequence could reduce the SLIM1 protein levels. In contrast, the truncated SLIM1 protein produced in the *slim1_KO* mutant contains only the C-terminal part and is unable to bind to the DNA (Supplementary Figs S2, S3A), although it clearly shows auto-activation of the reporter gene expression in yeast two-hybrid assays, suggesting the existence of a transcriptional activation domain in the C-terminal portion of SLIM1 (Supplementary Fig. S3B). Interestingly, the C-terminal location of the activation domain was also suggested for maize ZmEIL1 protein (Shi *et al.*, 2017). Unexpected results were obtained from metabolite analyses of the *slim1_KO* mutant. While our data for seedlings grown in normal sulfur supply support some data obtained for other *slim1* mutants concerning lower sulfate and glutathione contents, the increased OAS level was never observed (Maruyama-Nakashita *et al.*, 2006; Yamaguchi *et al.*, 2020; Jobe *et al.*, 2021). A possible explanation is that the *slim1_KO* mutant assimilates less sulfate than needed while still synthetizing OAS due to the malfunctioning control mechanism, and though the cysteine pool is not affected, probably to keep the appropriate translation level, the major storage form of cysteine, glutathione, is severely reduced. It is not in accordance with the known regulatory link between OAS and the high-affinity uptake system for sulfate (Takahashi *et al.*, 2011); however, we cannot exclude the involvement of SLIM1 in such regulation. The unexpected findings of increased sulfate, cysteine, and glutathione pools during the growth of the *slim1_KO* mutant in dS conditions could be explained by its highly reduced size. Such an observation was already made for some mutants in cysteine synthesis, which responded with decreased growth and translation to maintain the cysteine steady-state level in leaves (Dong *et al.*, 2017). It is, however, different from the other *slim1* mutants, which tend to further decrease sulfate, cysteine, and glutathione pools in dS conditions, pinpointing again the distinctness of the *slim1_KO* mutant (Maruyama-Nakashita *et al.*, 2006; Dietzen *et al.*, 2020). We also evaluated the expression of *SLIM1* at the transcriptional level using RT-qPCR primers annealing to the 3ʹ-terminus of the transcript (present in the *slim1_KO* mutant) (Fig. 3A). Surprisingly, the *SLIM1* expression level was largely diminished in the *slim1_KO* mutant suggesting that such truncated transcript may be less stable and therefore prone to degradation. This observation again underlines that *slim1_KO* is a knock-out mutant.

The most impressive phenotype of *slim1* mutants is the inhibition of germination by glucose during dS conditions. Environmental factors, such as light, temperature, water, and nutrients, primarily regulate seed germination via the metabolism and signaling pathways of gibberellin, and abscisic acid (ABA), with ABA promoting and maintaining dormancy and gibberellin promoting germination (Finkelstein *et al.*, 2008; Shu *et al.*, 2016). High glucose concentrations have been shown to delay germination in a variety of plants, but the mechanisms behind this have yet to be fully understood (Dekkers *et al.*, 2004; Zhao *et al.*, 2009; Zhu *et al.*, 2009). Exogenously applied high glucose concentrations during germination result in increased ABA biosynthesis (Price *et al.*, 2003) and inhibition of genes associated with ABA catabolism (Zhu *et al.*, 2009). However, high glucose rather than increasing cellular ABA levels leads to a delay in its degradation. Furthermore, the glucose-induced germination delay appears to be independent of hexokinase (Price *et al.*, 2003; Dekkers *et al.*, 2004). It has been shown recently, that nutrients, such as nitrate, can stimulate germination, even in the presence of high glucose concentrations, by enhancing the expression of some of the ABA biosynthesis and signaling genes (Ikeya *et al.*, 2020). It was also reported that sulfate supply affects the synthesis and steady-state levels of ABA in Arabidopsis (Cao *et al.*, 2014). Additionally, ABA can up-regulate the transcript level of some S-metabolism-related genes, which are partly regulated by SLIM1. This might suggest that ABA may have a stimulating effect on SLIM1, and hence the observed phenotype of the germination delay of *slim1* mutants in high glucose.

Expression of ‘OAS cluster’ genes is further inducible by sucrose under sulfur deficiency, indicating that sucrose controls the level or activity of transcription factors. It has been found that sucrose may control translation efficiency, affect the recruitment of ribosomes to specific mRNAs, and control the stability of the mRNA and proteins (Wang *et al.*, 2020; Yoon *et al.*, 2021). Plausibly sucrose may increase the degradation of a repressor protein by stimulating interaction between the repressor and E3 ubiquitin ligase, or it can prevent the degradation of SLIM1. It has been shown that the SLIM1 protein level is controlled by proteasomal degradation (Wawrzynska and Sirko, 2020). In Arabidopsis, HXK1 promotes the proteasome-dependent degradation of EIN3 and EIL1 through the C-terminus, which is stabilized by ethylene. Consistent with this, the *ein3* mutant is hypersensitive to glucose but insensitive to ethylene (Yanagisawa *et al.*, 2003; Yoo *et al.*, 2008). There are two F-box proteins, EIN3 BINDING F-BOX 1 and 2 (EBF1 and EBF2), that specifically recognize EIN3 to mark this protein with ubiquitin in the absence of an ethylene signal (Guo and Ecker, 2004). Recently, it was demonstrated that EBF1 can also bind to SLIM1, although the importance of that interaction is not clear (Wawrzynska and Sirko, 2020). If EBF1 serves for the degradation of SLIM1, for example during sulfur sufficient conditions, the degradation of EBF1 during ethylene emission might have a stabilizing effect not only on EIN3 but also on SLIM1 (Konishi and Yanagisawa, 2008). It has been shown that during sulfur deficiency ethylene emission increases, and thus SLIM1 degradation may be terminated through that signal (Moniuszko *et al.*, 2013). Another layer of complexity is added by the fact that EIN3 and SLIM1 can form heterodimers to outcompete SLIM1 from its targeted DNA-binding sites (Wawrzynska and Sirko, 2016). Because the level of EIN3 is negatively controlled by the glucose signal, it is quite plausible that degradation of EIN3 frees SLIM1 from heterodimers, allowing for DNA interaction.

It is noticeable that the 5% mannitol that served in our experiments as an osmotic control to glucose treatment exerted a comparable effect on ‘OAS cluster’ gene expression. Similarly, *slim1_KO* seedlings exposed to 5% mannitol displayed a lowered germination ability. Almost all abiotic stressors, including osmotic stress, cause oxidative damage in plant cells and result in the generation of reactive oxygen species (Upadhyaya *et al.*, 2013). However, plants have evolved mechanisms to reduce their oxidative damage, including the accumulation of antioxidant compounds, such as ascorbic acid, thioredoxin, and glutathione. *slim1* mutants have lower glutathione levels in shoots than the wild-type, confirming the role of SLIM1 in sulfate metabolism and thiol synthesis (Fig. 1C) (Maruyama-Nakashita *et al.*, 2006; Yamaguchi *et al.*, 2020; Jobe *et al.*, 2021). The sensitivity of the *slim1_KO* mutant to the high mannitol in normal sulfur supply that we observed (Fig. 2B) can therefore be explained by decreased thiol production resulting in increased oxidative stress. Interestingly, another link between osmotic stress and sulfur metabolism was revealed recently (Zhao *et al.*, 2020). H_2_S, arising from cysteine hydrolysis, alleviates the osmotic stress by elevating phospholipase D activity and suppressing reactive oxygen species by regulating antioxidant enzyme activities and increasing their gene expressions in Arabidopsis. It is therefore plausible that *slim1* mutants with the lowered glutathione pool (the storage form of cysteine) cannot produce enough H_2_S to cope with osmotic stress, hence the observed sensitivity to mannitol.

There is a question as to which of the sugars is a signaling molecule during sulfur deficiency. For example, sucrose, the primary transport sugar in plants, can be sensed as a signal directly (Chiou and Bush, 1998) or, alternatively, a signal can be generated by its cleavage products, glucose or UDP-glucose and fructose (Sheen, 2014). In contrast, a significant amount of glucose fed to plants can rapidly be converted to sucrose as observed in detached tobacco leaves 8 h after glucose feeding (Yoon *et al.*, 2021). Sucrose signaling mechanisms are mostly unexplored; however new data suggest that sucrose-specific functions exist in plants (Yoon *et al.*, 2021). Expression of ‘OAS cluster’ genes in Arabidopsis is specifically induced by external sucrose while glucose is less effective. Interestingly, the induction of these genes by short-term sulfur starvation was affected in the *hxk1* mutant, indicating that HXK1 is positively involved in that process.

Sugar production and signaling pathways are intricately related to other regulatory pathways to coordinate nutrient distribution and use, as well as growth and development. For instance, genes involved in carbon, nitrogen, and sulfur metabolism were regulated by glucose (Price *et al.*, 2004; Miao *et al.*, 2016). An intriguing result is that some glucose-responsive genes are also partly controlled by SLIM1 as their transcription is affected in the *slim1_KO* mutant (Fig. 3A). The transcriptional inhibition of ethylene biosynthesis and perception is an early event during glucose signaling. Several ethylene biosynthetic and signaling genes as well as both *EIN3* and *EIL1* were repressed by glucose (Price *et al.*, 2004). *SLIM1* was not on the list of genes with changed transcription due to glucose treatment. However, in the conditions of our experiment glucose treatment induced *SLIM1* transcription irrespective of the sulfur status in the environment (Fig. 3A), implying its involvement in plant glucose response. Recently, a new analysis of the published data revealed that the core genes regulated by SLIM1 are enriched in the category of sugar transporters, suggesting that SLIM1 might be a link to regulating sugar homeostasis during sulfur deficiency (Ristova and Kopriva, 2022). Multiple glucose signal transduction pathways exist in plants. There are at least three distinct pathways, according to the expression patterns of 26 genes involved in various cellular functions: (i) a HXK-dependent pathway, (ii) a HXK enzymatic activity-dependent pathway, and (iii) a HXK-independent pathway (Xiao *et al.*, 2000). We assayed the expression of genes belonging to each of the three pathways and found that SLIM1 participates in glucose signaling via HXK1, especially during sulfur deficiency. The molecular aspects of HXK1 involvement in translating the glucose signal to transcriptional machinery are still unresolved. It was revealed that HXK1 forms a glucose signaling complex core with two nuclear proteins, the vacuolar H^+^-ATPase B1 (VHA-B1) and the 19S regulatory particle of proteasome subunit (RPT5B), and directly controls the transcription of specific target genes independent of glucose metabolism (Cho *et al.*, 2006). Among others, this complex was shown to bind to the promoter of *CAB2* gene. The repression of *CAB2* by glucose seems to be also dependent on SLIM1 (Fig. 3A). In dS conditions, the absence of SLIM1 negatively affects the expression of *CAB2* even without glucose. It is therefore highly probable that SLIM1 may form complexes with other transcriptional factors.

It seems that the order of applied stressors is very important to observe the SLIM1-dependent glucose signaling event. In seedlings starved for 7 d of sulfur, the addition of high glucose for 17 h has no impact on either *SDI1* or *LSU1* gene expression, while we observed the enhancement of both transcript levels in short-term sulfur-starved seedlings grown on high glucose from germination. The observed effect is puzzling especially for *SDI1* expression. SDI1 is a negative regulator of the MYB28 transcription factor that plays a major role in the induction of aliphatic glucosinolate biosynthetic genes (Aarabi *et al.*, 2016). In contrast, glucose was found to have a beneficial effect on the *MYB28* transcript level and hence glucosinolate biosynthesis (Miao *et al.*, 2013). It was also found recently that glucose can be liberated from glucosinolates during the night to provide a carbon source for primary metabolism (Morikawa-Ichinose *et al.*, 2020). Therefore, it is tempting to speculate that in the conditions of short-term sulfur limitation and high glucose, plants enhance the level of SDI1 to prioritize usage of sulfate for primary metabolites rather than storing it in glucosinolates. We do not see the effect of glucose in seedlings starved long-term for sulfur as the positive impact of glucose signaling is stopped in such conditions to prevent a further reduction of the primary sulfur metabolism. This effect is similar to the response of the *ein3* mutant to sulfur deficiency (Wawrzynska and Sirko, 2016). In seedlings starved for sulfur short-term, the mutant clearly showed increased induction of SLIM1-dependent genes in comparison with the wild-type, while during prolonged sulfur starvation this effect was alleviated with the *ein3* mutant showing similar expression levels to the wild-type. This underlines that SLIM1 probably integrates many signals and, depending on the severity and duration of sulfur starvation, it translates them adequately to the transcriptional machinery.

A very interesting effect of SLIM1’s action was observed for *LSU1* gene expression. During sulfur deficiency SLIM1 is certainly a transcriptional activator of *LSU1*; however, this role is not conserved and it may also have a negative effect on *LSU1* transcription under sulfur sufficiency. In the *slim1_KO* mutant, we observed a higher level of *LSU1* transcript compared with the wild-type in sulfur sufficient conditions. A negative role of SLIM1 in ‘OAS cluster’ gene expression regulation was already observed during arsenic treatment (Jobe *et al.*, 2021). Such dual, both activator and repressor, abilities were reported for the close homolog of SLIM1, the transcription factor EIN3, which can either act alone or cooperate with other transcription factors (Solano *et al.*, 1998; Wawrzynska and Sirko, 2016; X. Q. Liu *et al.*, 2017; Zheng *et al.*, 2017; Meng *et al.*, 2020; Xiao *et al.*, 2021; Zhao *et al.*, 2021). Notably, EIN3 was found to bind to the promoter sequence and repress the expression of *LSU1* under cadmium stress (Kong *et al.*, 2018). As such, EIN3 emerges as a major integrator of external and internal signals that regulates seedling growth and development in an adaptable manner. We might also presume a similar role for SLIM1, which might control the transcriptional response to different signals, especially in sulfur limiting conditions.

**Fig. 7.**
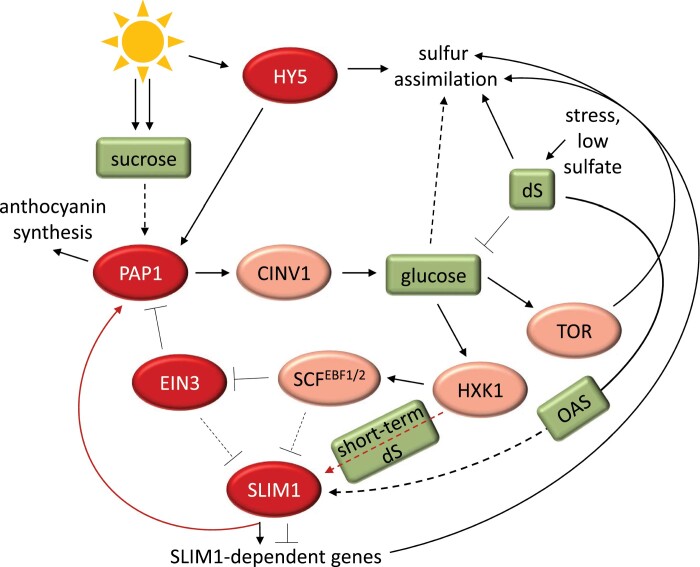
Model of sugar and sulfur deficiency sensing and signaling in Arabidopsis. Glucose can be either transported into the cell or mobilized from sucrose by cytosolic invertase (CINV) positively regulated by the PAP1 transcription factor (Meng *et al.*, 2020). PAP1, which also activates anthocyanin biosynthesis in the presence of sucrose (Kwon *et al.*, 2011), is inhibited at the transcriptional level by EIN3 but induced by HY5 in light and SLIM1 in sulfur deficiency (dS) (Shin *et al.*, 2013; Meng *et al.*, 2020). Hexokinase (HXK1) activated by glucose negatively affects EIN3 stability (Yanagisawa *et al.*, 2003), and it additionally stimulates SLIM1 to control selected gene expression during dS (Maruyama-Nakashita *et al.*, 2006). SLIM1 is putatively activated in dS via enhanced OAS concentration (Hubberten *et al.*, 2012). dS diminishes glucose level and affects glucose–TOR signaling (Dong *et al.*, 2017). TOR, in addition to glucose and light (acting through the transcription factor HY5), induces the transcription of sulfate assimilation genes (Lee *et al.*, 2011; Dong *et al.*, 2017). EIN3 may negatively impact SLIM1 binding to DNA (Wawrzynska and Sirko, 2016) while its abundance is negatively controlled by E3 ubiquitin ligases EBF1/2 (Guo and Ecker, 2004). EBF1 can also bind to SLIM1 (Wawrzynska and Sirko, 2020). Transcription factors are shown in red ovals, while other proteins are shown in pink ovals. Arrows and bars represent positive and negative regulation, respectively. Solid lines indicate direct regulation, and dotted lines indicate either indirect regulation or regulation in an unknown manner. The lines in red are supported by experimental data provided by this study. For more explanation, see the main text.

The ability of SLIM1 to bind to the *PAP1* promoter and positively regulate its transcription is a novel finding. In contrast, EIN3 is a negative regulator of *PAP1* transcription (Meng *et al.*, 2020). This adds another layer to the SLIM1–EIN3 interplay. Under sulfur deficiency conditions, SLIM1 seems to be crucial for inducing the production of anthocyanins, which are protective compounds for chlorophylls and the carotenoids and the signal to slow down photosynthesis. Anthocyanin accumulation in plants is often associated with nutritional deficiencies, particularly nitrogen, phosphorus, and sulfur, as a strategy to avoid over-accumulation of carbohydrates in tissues and to prevent physiological problems (Belwal *et al.*, 2020). This is fundamental for coupling the rate of metabolism of the macronutrients with photosynthesis. This finding also brings up another element in the discussion—diurnal regulation of sulfur metabolism. Sulfate assimilation and glucosinolate biosynthesis pathways are regulated by light (Huseby *et al.*, 2013). LONG HYPOCOTYL5 (HY5), a bZIP transcription factor and a positive regulator of photomorphogenesis (Ang *et al.*, 1998), directly binds to the promoters of >1000 light-inducible genes, including *APR* and the gene for one of the main sulfate transporters, *Sultr1;2* (J. Lee *et al.*, 2007; B. R. Lee *et al.*, 2011). Additionally, OAS treatment triggered the HY5-dependent induction of *APR*. Interestingly, HY5 acts as a repressor but also an activator of selected transcription factors from the glucosinolate biosynthesis pathway, although not through direct binding to their promoters (Huseby *et al.*, 2013). However, it has been demonstrated that HY5 also directly binds to the *PAP1* promoter to activate the biosynthesis of anthocyanin (Shin *et al.*, 2013). Notably, SLIM1-dependent activation of the ‘OAS cluster’ genes also fluctuates in a diurnal manner following the internal changes in OAS concentration with a clear increase during the night (Hubberten *et al.*, 2012). In this context, the involvement of SLIM1, EIN3, and HY5 in the regulation of plant metabolism seems to be more complex (Fig. 7). There must be a mechanism for the integration of different signals during sulfur deficiency and fine-tuning transcriptional and other responses. Dissection of the role of each individual transcription factor, their interplay, and the hierarchy in the control of sulfur metabolism regulation will thus be an exciting topic for further research.

### Conclusions

In summary, we demonstrate here that the SLIM1 transcription factor is involved in sugar signaling during sulfur deficiency. Our results suggest that SLIM1 acts together with HXK1 to fine-tune gene expression in response to sulfur limitation but in accordance with plant photosynthetic capacity. The sensitivity of the *slim1_KO* mutant to high glucose implies that the glucose signal during sulfur deficiency is transmitted through SLIM1 to its responsive genes. This interplay seems to be especially important during the initial steps of the response to sulfur deficiency. Interestingly, we found that SLIM1 also binds to the *PAP1* promoter to induce the production of anthocyanin and slow down the photosynthesis rate and thus sugar production during sulfur deficiency. Taken together, our data support a model in which SLIM1 acts downstream of HXK1 to integrate the signals of sulfur status with the plant’s energetic state. SLIM1, therefore, arises as an important hub for collecting different metabolic signals. The perception and transmission of these metabolic cues contribute to the regulation of plant growth and development through intertwined signaling networks.

## Supplementary data

The following supplementary data are available at *JXB* online.

Fig. S1. *TUA3* is a stable reference gene.

Fig. S2. CRISPR/Cas-guided deletion of SLIM1 in the *slim1_KO* mutant.

Fig. S3. Y1H and Y2H screens of the chimeric SLIM1 protein from *slim1_KO* mutant.

Fig. S4. The phenotype of *slim1_KO × prSLIM1::SLIM1* lines in nS and dS conditions.

Fig. S5. Phenotypes of *slim1-2* and SALK_089129 lines under mannitol/glucose treatment(s).

Fig. S6. Phenotypes, anthocyanin content, and *PAP1* expression in *slim1-2* and SALK_089129 lines under sucrose treatment.

Table S1. List of oligonucleotide primers and constructs used in this study.

Table S2. Gene-specific primers used for qRT-PCR.

Table S3. Metabolite contents in the shoots of the wild-type and the *slim1_KO* mutant.

erac371_suppl_Supplementary_Figures_S1-S6_Tables_S1-S3

## Data Availability

Raw data that support the findings of this study are available from the corresponding author, upon reasonable request.

## Abbreviations

dS: sulfur deficient
nS: normal sulfur
OAS: *O*-acetylserine
qRT-PCR: quantitative real time PCR
