# Supplementary material for: The SLIM1 transcription factor affects sugar signaling during sulfur deficiency in Arabidopsis
Source: J Exp Bot. 2022 Sep 13;73(22):7362–79. doi: 10.1093/jxb/erac371 (PMC9730805; doi:10.1093/jxb/erac371)
Supplement: erac371_suppl_Supplementary_Figures_S1-S6_Tables_S1-S3 [file erac371_suppl_supplementary_figures_s1-s6_tables_s1-s3.pdf]

**A**

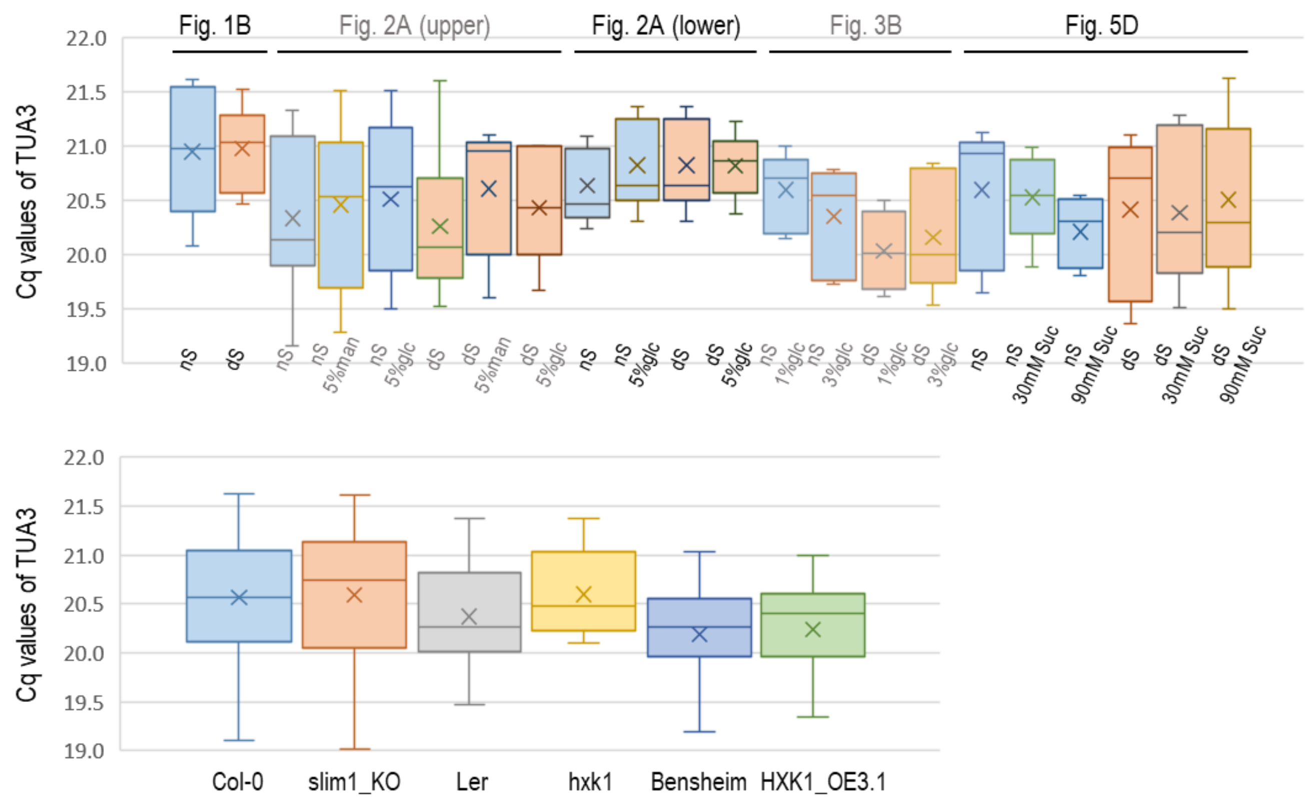


**B**

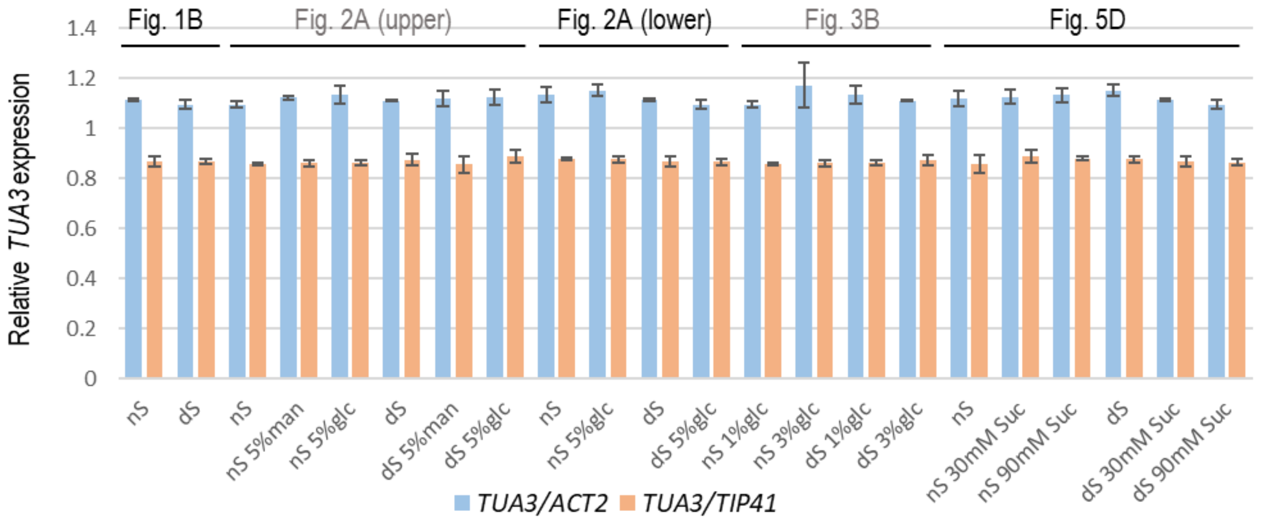


**Supplementary Figure S1.** *TUA3* is a stable reference gene.

**A.** Quantification cycle (Cq) value of *TUA3* reference gene across all the experimental samples. In the upper panel the experimental conditions of the study while in the lower panel different plant genotypes used in the study. The line across the box depicts the median while the cross the mean value. Whiskers represent the local maximum and minimum values. The box indicates the 25% and 75% percentiles.

**B.** Test of the internal control *TUA3* stability by *ACTIN2* and *TIP41* (by RT-qPCR). Mean Cq value of *TUA3* for each treatment was divided by mean Cq value of either *ACT2* or *TIP41* for the same treatment. The expression of *TUA3* did not change significantly (P>0.05, Student's t-test) under all experimental conditions in this study (compared to control conditions (nS) in each experiment).

**A**

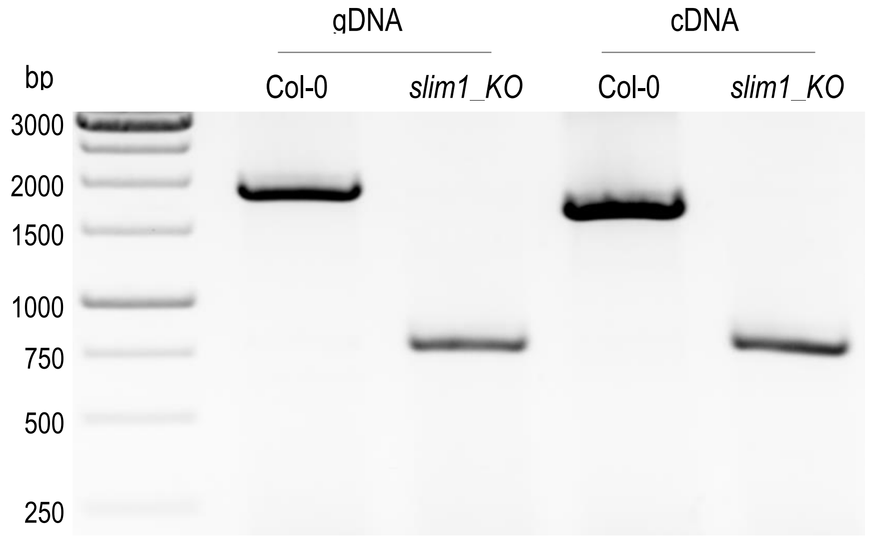


**B**

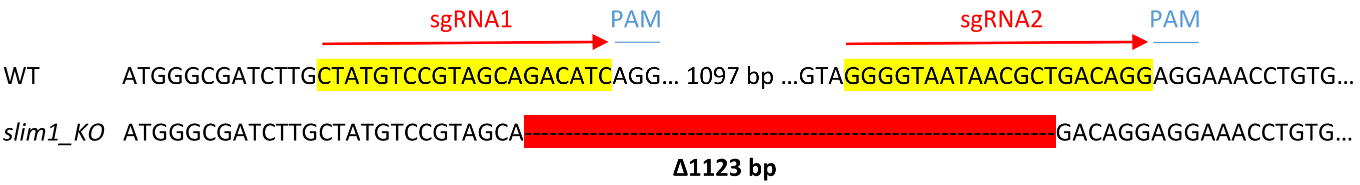


**SLIM1 protein sequence in *slim1_KO* mutant:**

MGDLAMSVADRRKPVVNSDSDYDVDGTEEASGSVSSKDSRRNQIQKEQPTAISHSVRDQDKAEKHRRRKRPRIRSGTVNRQEEEQPEAQQRNILPD
MNHVDAPLLEYNINGTHQEDDVVDPNIALGPEDNGLELVVPEFNNNYTYLPLVNEQTMMPVDERPMLYGPNPNQELQFGSGYNFYNPSAVFVHN
QEDDILHTQIEMNTQAPPHNSGFEEAPGGVLQPLGLLGNEDGVTGSELPQYQSGILSPLTDLDFDYGGFGDDFSWFGA

**C**

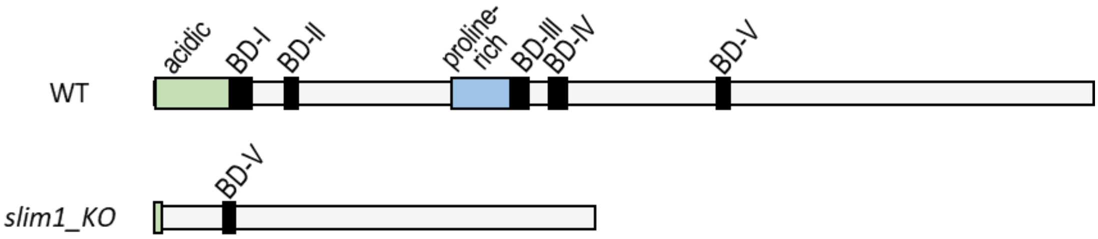


**Supplementary Figure S2.** CRISPR/Cas-guided deletion of SLIM1 in *slim1_KO* mutant.

**A.** PCR analysis of the *SLIM1* encoding sequence in the wild type (Col-0) and *slim1_KO* mutant on the genomic and transcriptomic level. *SLIM1* was amplified with primers framing entire coding sequence (used for SLIM1 cloning, Table S1); expected sizes: gDNA for *SLIM1* – 1930bp, gDNA for *SLIM1* in *slim1_KO* mutant – 807bp, cDNA for *SLIM1* – 1704bp, cDNA for *SLIM1* in *slim1_KO* mutant – 807bp.

**B.** Dual sgRNA-based targeted 1123bp-deletion of genomic region of At1g73730 results in the 'in frame' deletion of 300aa starting from the 10$^{th}$ residue of SLIM1 protein.

**C.** Schematic structural diagram of SLIM1 protein in the wild-type and *slim1_KO* mutant. Acidic regions, proline-rich regions, and basic domains (BD) are represented by different colored boxes.

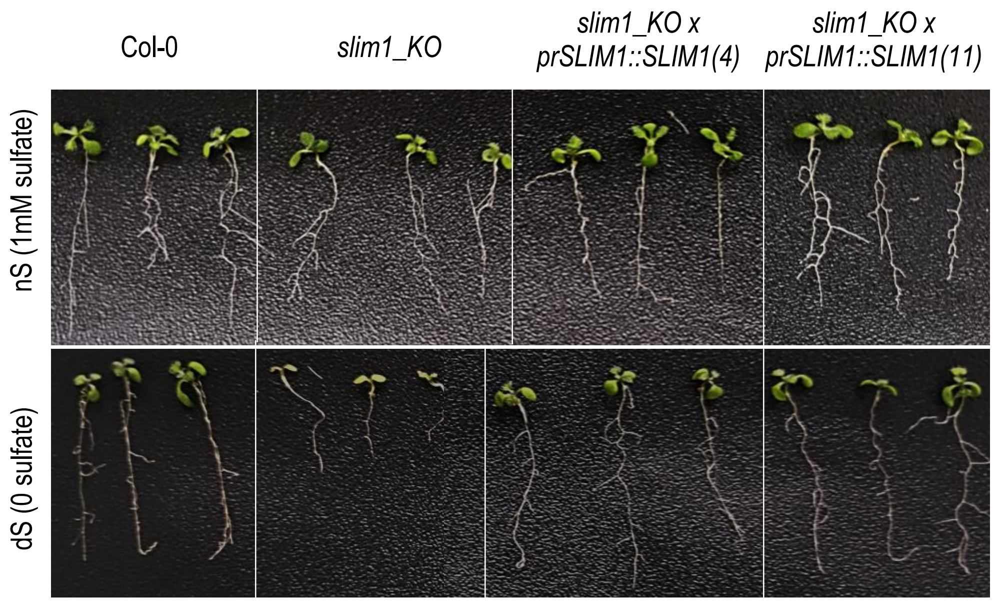


**Supplementary Figure S3.** The phenotype of *slim1_KO* x *prSLIM1::SLIM1* lines in nS and dS conditions.

Growth phenotype of wild-type (Col-0), *slim1_KO* mutant and two other independent *slim1_KO* x *prSLIM1::SLIM1 lines* after 12 d in normal sulfate supply (nS) and no sulfate (dS) conditions.

A

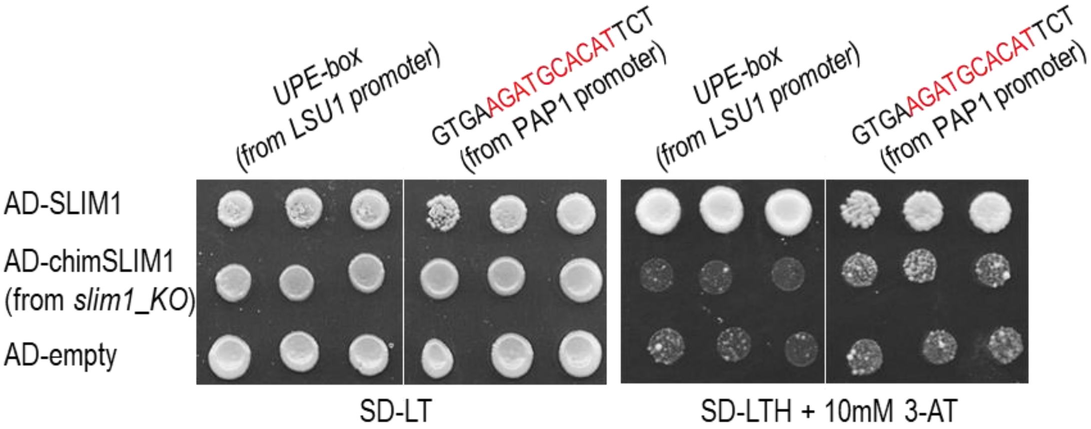


B

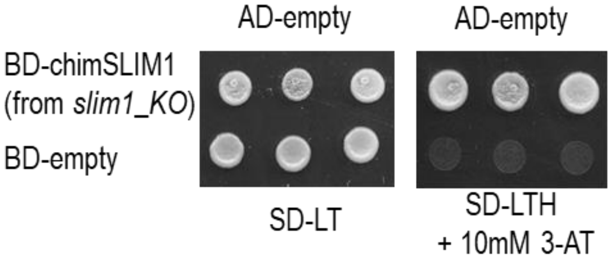


**Supplementary Figure S4.** Y1H and Y2H screens of the chimeric SLIM1 protein from *slim1_KO* mutant.

**A.** Yeast one-hybrid screening. Growth of yeast harboring *HIS3* reporter fused either to DNA fragment from *LSU1* promoter containing SLIM1 binding site (UPE-box) (Wawrzyńska et al., 2010) or to putative SLIM1 binding site from *PAP1* promoter and co-transformed with either the SLIM1 or chimeric SLIM1 cloned from *slim1_KO* mutant. Yeasts were grown for 3 d at 30°C on a selective medium lacking leucine, tryptophan, and histidine with either 10 mM 3-aminotriazole and photographed. On the left-hand side, yeast growth on the control medium lacking leucine and tryptophan is shown. Three independently transformed yeast colonies are shown.

**B.** Yeast two-hybrid screening. Growth of yeast transformed with chimeric SLIM1 cloned from *slim1_KO* mutant fused with GAL4 BD and empty AD vector. Yeasts were grown for 3 d at 30°C on a selective medium lacking leucine, tryptophan, and histidine with either 10 mM 3-aminotriazole and photographed. On the left-hand side, yeast growth on the control medium lacking leucine and tryptophan is shown. Three independently transformed yeast colonies are shown.

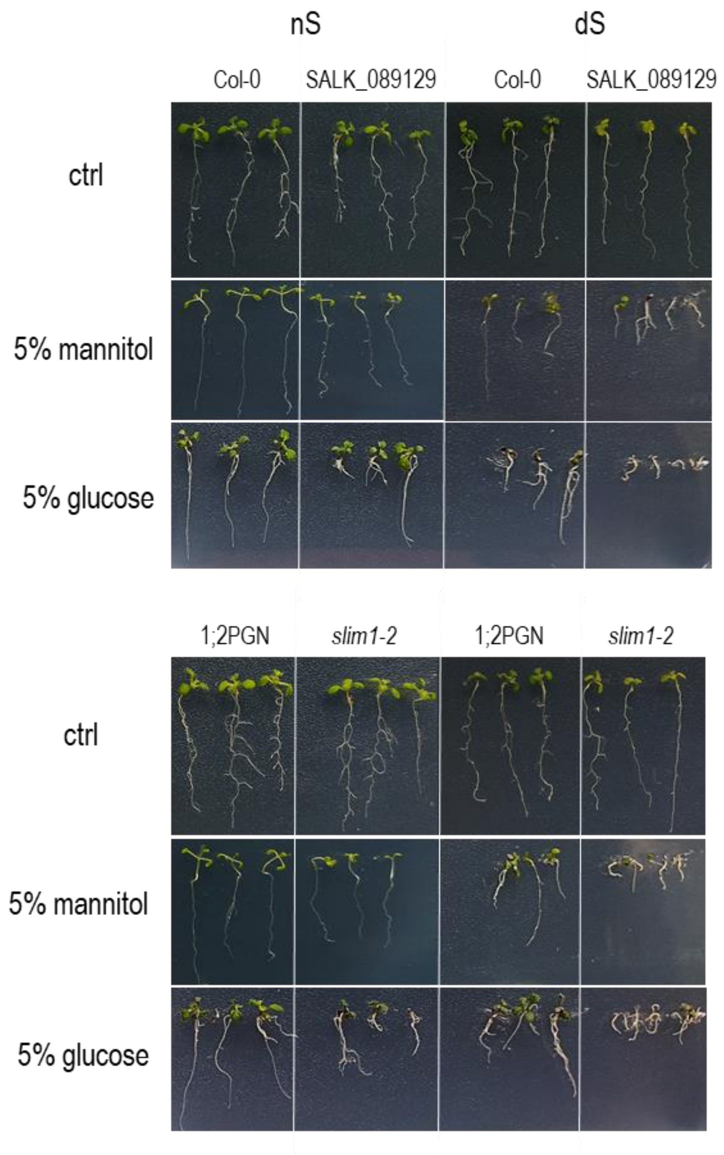


**Supplementary Figure S5.** Phenotypes of *slim1-2* and SALK_089129 lines under mannitol/glucose treatment(s).

Growth phenotype of seedlings grown 12 days in the presence of either 5% glucose or 5% mannitol (osmotic control) and different sulfate availability (normal sulfate supply (nS) and sulfate deficiency (dS)). 1;2PGN and Col-0 are parental lines for *slim1-2* and SALK_089129 lines, respectively.

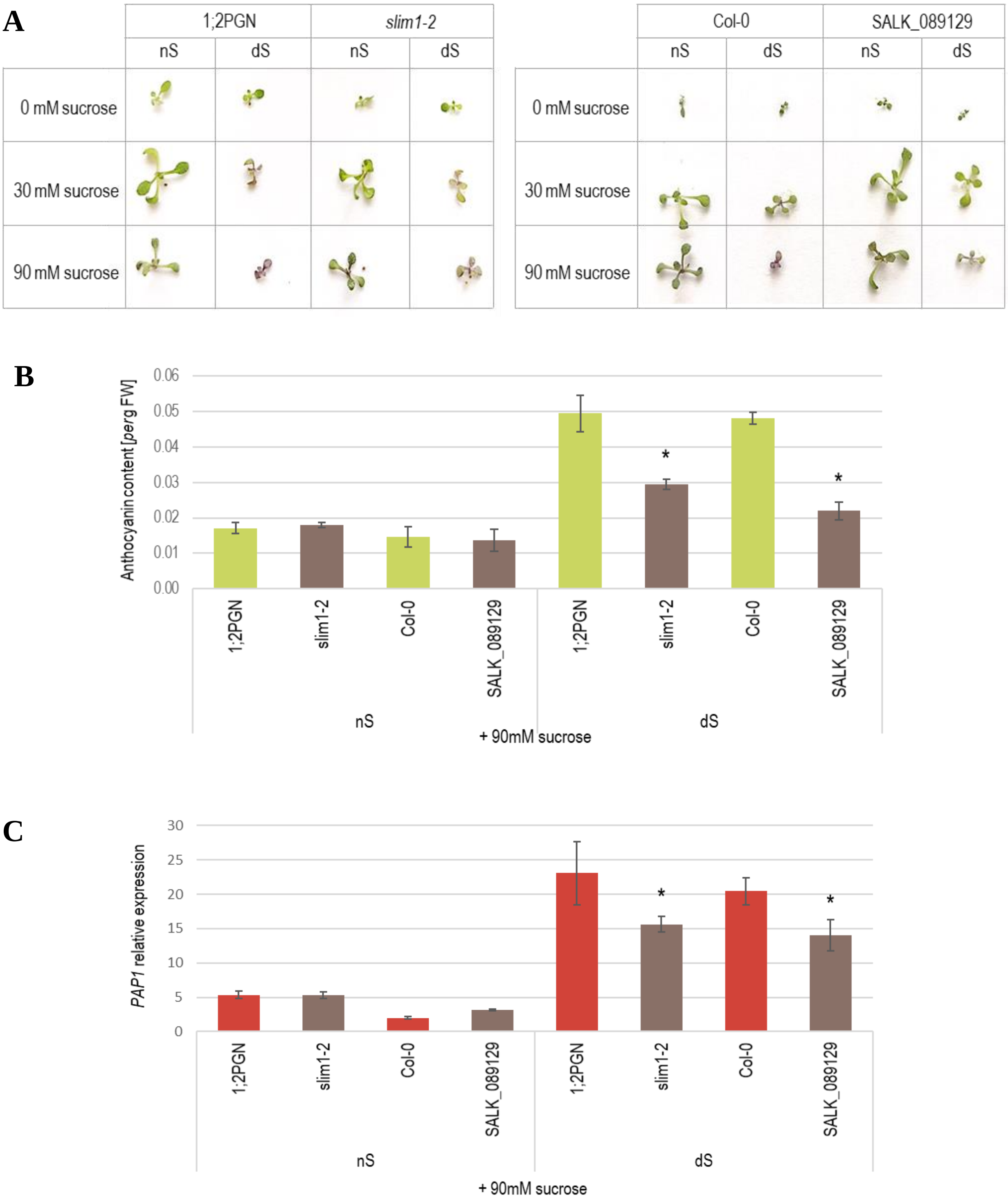


**Supplementary Figure S6.** Phenotypes, anthocyanin content, and *PAP1* expression in *slim1-2* and SALK_089129 lines under sucrose treatment.

**A.** Growth phenotype of seedlings grown 12 days in the presence of increased sucrose concentration and different sulfate availability (normal sulfate supply (nS) and sulfate deficiency (dS)). 1;2PGN and Col-0 are parental lines for *slim1-2* and SALK_089129 lines, respectively.

**B.** Anthocyanin content in 12-day-old seedlings grown in the presence of 90 mM sucrose and different sulfate availability. The asterisks indicate statistically significant differences (t-Student test, $p < 0.05$) as compared to the corresponding parental line at the same treatment. The experiment was repeated three times.

**C.** Relative *PAP1* expression in 12-day-old seedlings grown in the presence of 90 mM sucrose and different sulfate availability. The asterisks indicate statistically significant differences (t-Student test, $p < 0.05$) as compared to the corresponding parental line at the same treatment. The experiment was repeated three times.

**Supplementary Table S1.** List of oligonucleotide primers and constructs used in this study.

| OLIGOS (5' - 3') | | VECTOR | CONSTRUCT | APPLICATION |
|---|---|---|---|---|
| For: | GCGAGCTCTCCAGAAGTAAATCATTAAGAACCAA | pHIS2.1 | prPAP1-HIS | bait for Y1H |
| Rev: | GCCGCGTGAACCCTCCATGGAACAAAG | | | |
| pPAP_UPE: | GCACGCGTGAAGATGCACATTCTGATCCACTAGTAATTCCTGGCAT | pHIS2.1 | pUPE-HIS | bait for Y1H |
| HISR: | GCGGATCCTCGGGGACACCAAATA | | | |
| pPAP_EIN: | GCACGCGTGACTTCAAATGCTTGTTGATCCACTAGTAATTCCTGGCAT | pHIS2.1 | pEIN-HIS | bait for Y1H |
| HISR: | GCGGATCCTCGGGGACACCAAATA | | | |
| For: | CACCATGGGCGATCTTGCTATGTC | pDEST_GADT7 | SLIM1 x pGAD | effector for Y1H |
| Rev: | AGCTCCAAACCATGAGAAATCA | | | |
| For: | CACCATGATGTTTAATGAGATG | pDEST_GADT7 | EIN3 x pGAD | effector for Y1H |
| Rev: | GAACCATATGGATACATCTTGCTGCT | | | |
| For: | CACCATGGGCGATCTTGCTATGTC | pDEST_GADT7 | chimSLIM1 (from slim1_KO) | effector for Y1H |
| Rev: | AGCTCCAAACCATGAGAAATCA | | | |
| For: | CACCATGGGCGATCTTGCTATGTC | pDEST_GBKT7 | chimSLIM1 (from slim1_KO) | effector for Y2H |
| Rev: | AGCTCCAAACCATGAGAAATCA | | | |
| For: | GCGAGCTCTCCAGAAGTAAATCATTAAGAACCAA | pGG | prPAP1 x GG | *PAP1* promotor activation *in planta* |
| Rev: | GCGGCCGCGAACCCTCCATGGAACAAAG | | | |
| For: | CTAGTGAAAATATAAAATCTAAAAACTGG | pGG | prPAP1m1 x GG | mutated *PAP1* promotor activation *in planta* |
| Rev: | CCAGTTTTTAGATTTTATATTTTCACTAG | | | |
| For: | TTAAGAAAAGACAAAAAATGCTTGTTAAAT | pGG | prPAP1m2 x GG | mutated *PAP1* promotor activation *in planta* |
| Rev: | ATTTAACAAGCATTTTTTGTCTTTTCTTAA | | | |
| For: | CACCATGGGCGATCTTGCTATGTC | pGWB420 | SLIM1 | effector for plant transformation |
| Rev: | AGCTCCAAACCATGAGAAATCA | | | |
| For: | CACCATGATGTTTAATGAGATG | pGWB420 | EIN3 | effector for plant transformation |
| Rev: | GAACCATATGGATACATCTTGCTGCT | | | |
| For: | CACCTTAGAGATGGCGAGAAGAAGC | pGWB419 | SLIM1 under native promoter | SLIM1 expression *in planta* |
| Rev: | AGCTCCAAACCATGAGAAATCA | | | |

**Supplementary Table S2.** Gene-specific primers used for qRT-PCR.

| AGI CODE | GENE NAME | OLIGOS (5' - 3') |
|---|---|---|
| At5g19770 | *TUA3* | For: GTATTGAACGCATCGTGTG |
| | | Rev: TGGGAGCTTTACTGTCTCGAA |
| At3g18780 | *ACT2* | For: GCACCAAGCAGCATGAAGATT |
| | | Rev: GGAACCACCGATCCAGACACT |
| At4g34270 | *TIP41* | For: GAATGGCTGGACAATGGAGTG |
| | | Rev: ATCAACTCTCAGCCAAAATCG |
| At1g73730 | *SLIM1* | For: TGACTTGGACTTTGACTATGGTG |
| | | Rev: CTATTGCCATGTCCTTTTGAACT |
| At5g48850 | *SDI1* | For: CAAATCTTTCCGTCCTCGTT |
| | | Rev: CAACTCAACTTGCTCCTCCA |
| At1g04770 | *SDI2* | For: CGAGCGAAGCATGTTCAGTT |
| | | Rev: CGTCAATGGCTTCTTCAGCTC |
| At3g49580 | *LSU1* | For: TTAAGTTGTGGCAGCGAACG |
| | | Rev: CCATGAGGAAGAGCATGCGA |
| At5g26220 | *GGCT1;2* | For: TGTTCGACATTGAGCATGAG |
| | | Rev: CGGTAAATCGAGTTGCTTCCT |
| At1g36370 | *SHM7* | For: TCCCAAATCTTCATCATCTTC |
| | | Rev: ATGCGTGTGAACAACCAAAC |
| At4g21990 | *APR3* | For: GGAATCCATTGTTGCTTCTGA |
| | | Rev: CCATGATCTCAAGAGGAGAA |
| At2g44460 | *BGLU28* | For: CGCGTTACGTTGCTCATATT |
| | | Rev: GAGCTGATGATCGGTTACGA |
| At1g56650 | *PAP1/MYB75* | For: TCCTAGAGGAAAGCCAAGAG |
| | | Rev: TCTCCATCGAAAAGACTCCAA |
| At4g29130 | *HXK1* | For: GCCTTTGAAGAGGATTGTGC |
| | | Rev: AAGCATCTTGAGTTTGCTACCAC |
| At1g29920 | *CAB2* | For: TCCATTCTCTGGCGAGTCAC |
| | | Rev: CTCGGGGTCAGCGGATAGA |
| At2g14610 | *PR1* | For: CATGGGACCTACGCCTACC |
| | | Rev: TTCTTCCCTCGAAAGCTCAA |
| At4g39210 | *APL3* | For: AGATCGGGAAAAACGTGGTG |
| | | Rev: GGCCTTTTCGACAACCACAG |

**Supplementary Table S3.** Metabolite contents in the shoots of the wild-type and the *slim1_KO* mutant.

| | nS | | dS | |
|---|---|---|---|---|
| | wild-type | *slim1_KO* | wild-type | *slim1_KO* |
| **sulfate** | 12.502 +/- 1.214 | 5.851 +/- 0.518 | 0.004 +/- 0.006 | 0.303 +/- 0.288 |
| **OAS** | 0.005 +/- 0.004 | 0.047 +/- 0.012 | 0.867 +/- 0.196 | 0.957 +/- 0.534 |
| **cysteine** | 25.802 +/- 1.622 | 27.871 +/- 5.461 | 16.449 +/- 4.768 | 38.763 +/- 0.921 |
| **glutathione** | 541.738 +/- 20.755 | 352.239 +/- 69.867 | 59.487 +/- 8.048 | 140.964 +/- 20.128 |

Plants were grown for 14 d in either normal sulfate supply (nS) or 10μM sulfate (dS) conditions. Only shoots were collected for measurements. In the table above the absolute values of each metabolite in nmoles/ mg FW (for sulfate and OAS) or pmoles/ mg FW (for cysteine and glutathione) with standard deviations.
